# Enhancing Photocathodic Performances of Particulate-CuGaS_2_-Based Photoelectrodes via Conjugation with Conductive Organic Polymers for Efficient Solar-Driven Hydrogen Production and CO_2_ Reduction

**DOI:** 10.1021/acsami.4c06083

**Published:** 2024-07-02

**Authors:** Tomoaki Takayama, Akihide Iwase, Akihiko Kudo

**Affiliations:** † Department of Applied Chemistry, Faculty of Science, Tokyo University of Science, 1-3 Kagurazaka, Shinjuku-ku, Tokyo 162-8601, Japan; ‡ Research Institute of Science and Technology, Carbon Value Research Center, Tokyo University of Science, 2641 Yamazaki, Noda-shi, Chiba-ken 278-8510, Japan

**Keywords:** particulate CuGaS_2_, conductive organic polymers, solar-driven hydrogen production, CO_2_ reduction, artificial photosynthesis

## Abstract

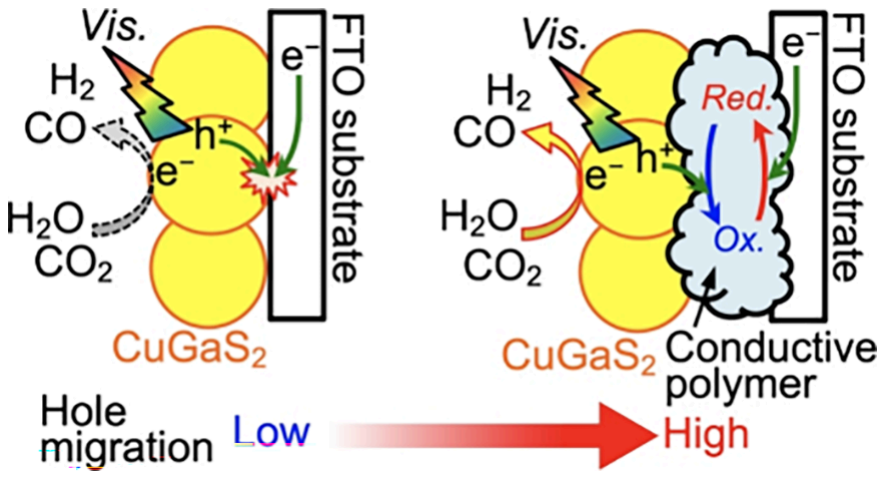

Modification with conductive organic polymers consisting of a thiophane- or pyrrole-based backbone improved the cathodic photocurrent of a particulate-CuGaS_2_-based photoelectrode under simulated solar light. Among these polymers, poly(3,4-ethylenedioxythiophene) (PEDOT) was the most effective in the improvements, providing a photocurrent 670 times as high as that of the bare photocathode. An incident-photon-to-current efficiency (IPCE) for water reduction to form H_2_ under monochromatic light irradiation (450 nm at 0 V vs RHE) was ca. 11%. The most important point is that modification of the conductive organic polymers does not involve any vacuum processes. This importance lies in the use of an electrochemically oxidative polymerization, not in a physical process such as vapor deposition of metal conductors. This is expected to be advantageous in the large-scale application of photocathodes consisting of particulate photocatalyst materials toward industrial solar-hydrogen production using photoelectrochemical-cell-based devices. Artificial photosynthesis of water splitting and CO_2_ reduction under simulated solar light was demonstrated by combining the PEDOT-modified CuGaS_2_ photocathode with a CoO_*x*_-loaded BiVO_4_ photoanode. Furthermore, how the cathodic photocurrent of the particulate-CuGaS_2_-based photocathode was drastically improved by the modification was clarified based on various characterizations and control experiments as follows: (1) selectively filling cavities between the particulate CuGaS_2_ photocatalysts and a conductive substrate (FTO; fluorine-doped tin oxide) with the polymers and (2) using a large driving force for carrier transportation governed by the polymers’ redox potentials adjusted by functional groups.

## Introduction

Photoelectrochemical water splitting and CO_2_ reduction using water as an electron donor have gathered much attention as a promising candidate for methodology to convert solar energy to chemical energy.^1−5^ Many efforts have been made to investigate photoelectrochemical properties of Cu-contained compounds, such as sulfides,^6−20^ selenides,^21−31^ and oxides.^32−35^ This is because most of the Cu-containing materials exhibit a *p*-type semiconductor character that is indispensable for employing them as photocathodes in photoelectrochemical reactions. Among them, it has been reported that Cu-contained metal-sulfide-photocatalyst powders are useful as photocathodes to reduce H_2_O and CO_2_ to H_2_ and CO, respectively, under simulated solar light.^7−10,13−15,17,19^ Moreover, their band gaps correspond to visible light and can be flexibly controlled by the formation of solid solutions.^7−9,13,15,16,19,36−42^ Nevertheless, photoelectrochemical performances of the particulate-based photocathodes are mostly lower than those of the thin films made by vaper deposition^16,22,28,30^ and precursor coating followed by either sulfurization or selenization.^7,14,17,23,27,29^ A main reason why the performances of the particulate-based photocathodes are lower is considered to be insufficient contacts between the particulate photocatalysts and a conductive substrate such as FTO (fluorine-doped tin oxide). In other words, there are many cavities between the particulates and the FTO. A solution for this problem is a particle-transfer method. This method is effective to obtain higher performances of the particulate-based photocathodes. This is due to a tight contact between the particulates and a metal layer.^13,19,21,24,25^ However, most of the aforementioned methods require vacuum processes, whereas some methods demand the use of highly toxic H_2_S gas and Se vapor. In this context, it is indispensable to develop a methodology capable of improving the performances of the particulate-based photocathodes even under an ambient preparation condition.

Combination of the particulate photocatalysts with a conductive material is an effective strategy in improving contacts between the particulates and the substrates such as FTO. We have reported that a reduced graphene oxide (RGO) works as a solid-state electron mediator between particulate CuGaS_2_ photocatalysts and an FTO substrate, resulting in an improvement in a cathodic photocurrent under simulated solar light.^10^ Importantly, CuGaS_2_ is combined with RGO of a conductive material by just mixing it in methanol under ambient pressure and temperature. This approach is well broadened into other photocatalyst-based systems such as Z-scheme systems.^18,33,43−47^ Taking the Z-scheme system as an example is helpful to understand other approaches using such conductive materials without any vacuum processes. Specifically, it has been reported that mixing either the Au^48^ or ITO^49^ (indium tin oxide) of conductive particles with particulate photocatalysts is useful for improving carrier transportation between hydrogen-producing photocatalysts and oxygen-producing photocatalysts. However, all of these approaches cannot selectively fill their cavities with conductive materials. Therefore, the combination of the particulate photocatalysts with such a conductive material must still be investigated for further enhancing the performances of the particulate-based photocathodes.

**Figure 1:**
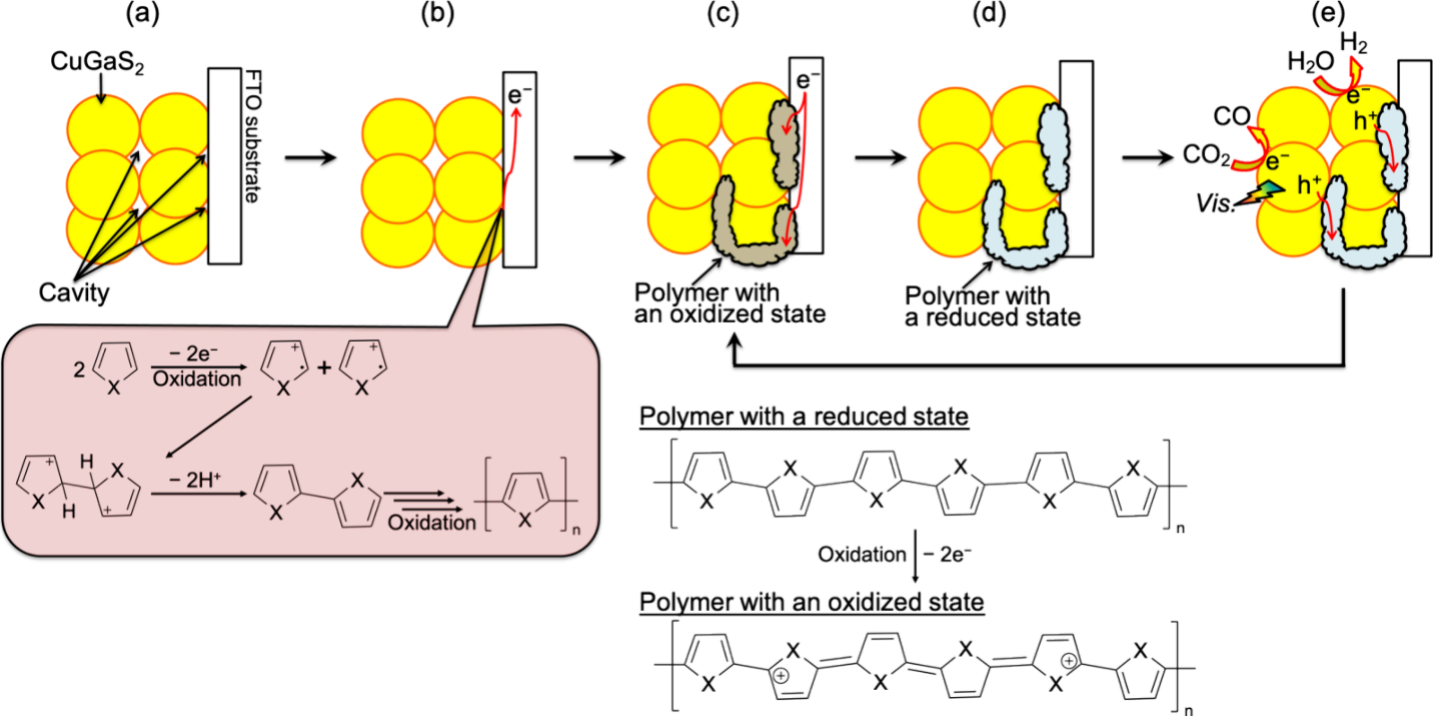
Proposed mechanism described in cross-section illustrations of particulate-CuGaS_2_-based photocathodes and the concept of the modification of conductive organic polymers: (a) pristine photocathode, (b) polymerization within the cavities, (c) electrochemical reduction of the deposited polymers, (d) formation of the reduced polymers, and (e) reoxidation of the reduced polymers by photogenerated holes.

Here, we focused on a series of *p*-type conductive organic polymers, polypyrrole, and poly(3,4-ethylenedioxythiophene). This is because these polymers possess electrochemical redox properties and can be polymerized on conductive substrates such as FTO by electrochemical oxidative coupling of their aromatic rings under ambient temperature and pressure.^50−53^ Our concept is concretely described in the following four steps as shown in Figure 1. First, the conductive polymer is selectively synthesized in the cavities between particulate CuGaS_2_ and an FTO substrate by a typical anodic dark current (Figures 1a and 1b). Second, the deposited polymer is electrochemically reduced around electronegative potentials in which cathodic photocurrents of particulate CuGaS_2_ are observed (Figures 1c and 1d). Third, the reduced state of the polymer returns to the oxidized state through an oxidation by holes photogenerated in the particulate CuGaS_2_ accompanied by H_2_O and CO_2_ reduction to form H_2_ and CO (Figure 1e). Finally, the oxidized polymer is again reduced by electrons supplied from an FTO substrate electrochemically (Figure 1c). Therefore, we expected that the polymer deposited in the cavities significantly boosts the hole transportation from particulate CuGaS_2_ to an FTO substrate.

In this study, effects of polymer modification on the photocathodic performance of the pariculate-CuGaS_2_-based photocathode were evaluated. This modification was performed under ambient pressure. Among these polymers, poly(3,4-ethylenedioxythiophene) (PEDOT) was the most effective in improving the performance of the particulate-based photocathode. The mechanisms were interpreted based on the structural and redox properties of the polymer-deposited photocathodes characterized by a scanning electron microscope, an X-ray photoelectron spectroscope, and a potentiostat. Furthermore, the CuGaS_2_ photocathode modified with PEDOT was successfully applied to artificial photosynthesis of water splitting and CO_2_ reduction by combining it with a CoO_*x*_-loaded BiVO_4_ photoanode. Herein, we display that the electrochemical polymer deposition approach overcomes the difficulty to selectively connect the particulate CuGaS_2_ photocatalysts to an FTO substrate, resulting in the comparable performance of the particulate-CuGaS_2_-based photocathode to that of thin-film photocathode made of CuGaS_2_.

## Experimental Section

### Preparation of a Particulate-CuGaS_2_-Based Photocathode

A powdered CuGaS_2_ photocatalyst was prepared by a conventional solid-state reaction.^9^ Cu_2_S (Kojundo Chemical; 99%) and Ga_2_S_3_ (Kojundo Chemical; 99.99%) were employed as the starting materials. These were well ground in the molar ratio of Cu:Ga = 1:1.1 using an agate mortar. The powdered mixture was sealed into a quartz ampule tube in a vacuum condition (<10^–1^ Pa) and sequentially heated at 1073 K for 10 h using a muffle furnace. The obtained yellow powder was identified to be a single phase of CuGaS_2_ by powdered X-ray diffraction (Rigaku; Miniflex, Cu Kα) (Figure S1). A particulate-CuGaS_2_-based photocathode was prepared by a drop-casting method. The CuGaS_2_ powder was carefully dispersed into ethanol (5–10 mg mL^–1^) by ultrasonication. The suspension was dripped on an FTO substrate that was cleaned beforehand with ozone. Ethanol of the suspension was dried at room temperature. The amount of the CuGaS_2_ powder accumulated on the FTO was almost 3 mg cm^–2^.

### Modification of Particulate-CuGaS_2_-Based Photocathodes with Conductive Organic Polymers

**Figure 2:**
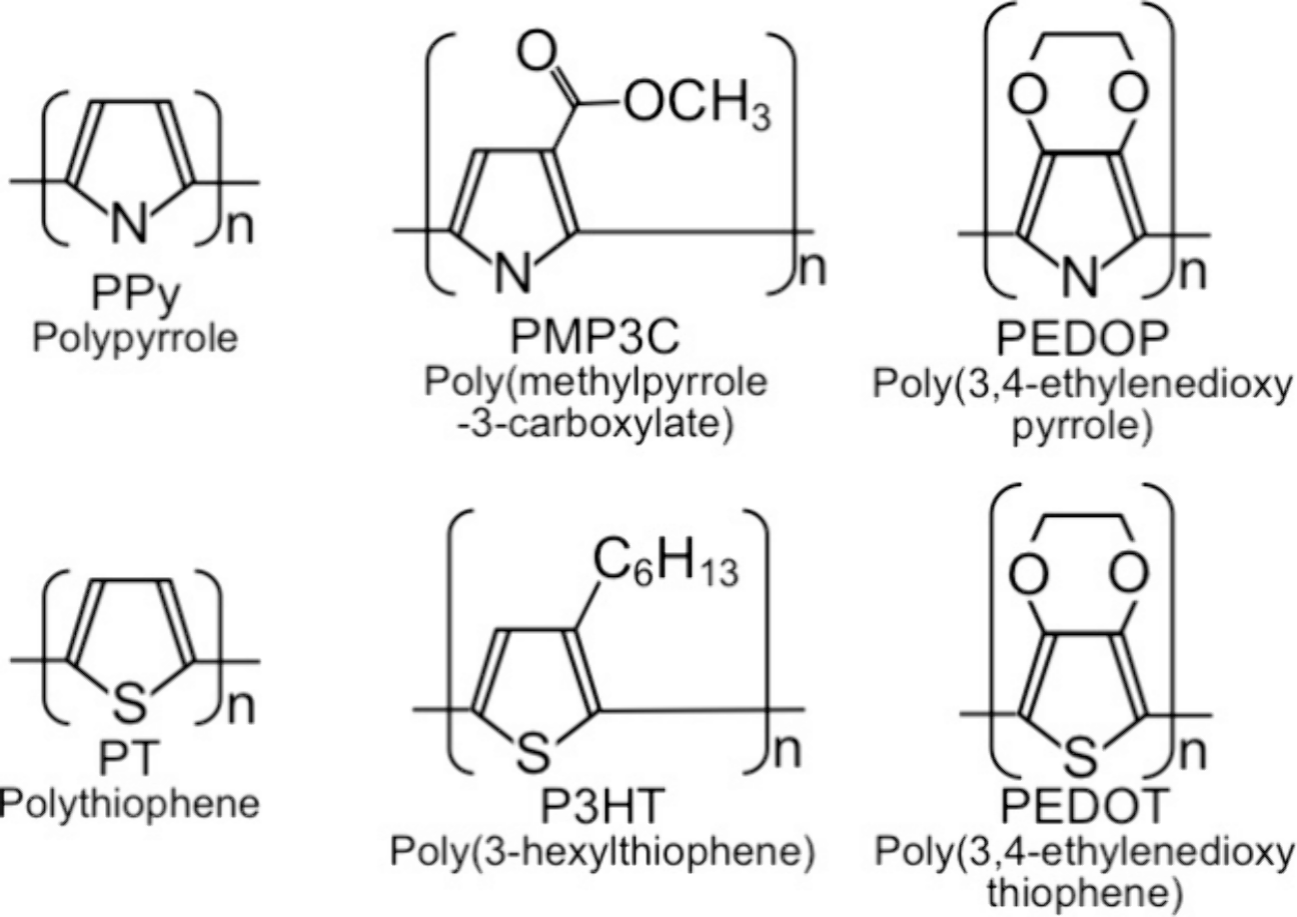
Polymers synthesized by an electrochemical oxidative polymerization method.

The polymers summarized in Figure 2 were synthesized by electrochemical oxidative polymerization. Typical anodic dark current of a particulate-CuGaS_2_-based photocathode was consumed for the polymerization. Pyrrole (Wako; 99.0%), methylpyrrole-3-carboxylate (Sigma-Aldrich; 97%), 3,4-ethylenedioxypyrrole (Sigma-Aldrich; 2% (w/v) in THF), thiophene (Sigma-Aldrich; 99%), 3-hexylthiophene (Sigma-Aldrich; 99%), and 3,4-ethylenedioxythiophene (Sigma-Aldrich; 97%) were employed as the monomers, resulting in the corresponding polymers described as PPy, PMP3C, PEDOP, PT, P3HT, and PEDOT, respectively. An acetonitrile solution containing both a monomer (0.2 mol L^–1^) and LiClO_4_ (0.1 mol L^–1^) were prepared in an ice bath (approximately 278 K), while the concentration of the 3,4-ethylenedioxypyrrole monomer was 4 mmol L^–1^. A particulate-CuGaS_2_-based photocathode of a working electrode was immersed in the mixture solution with an Ag/AgCl reference electrode and a counter electrode of a bare FTO substrate (Figure S2). The reference electrode was separated from the acetonitrile solution by using a typical KCl–agar salt bridge. The monomers were polymerized in mainly cavities between particulate photocatalysts and an FTO substrate by applying electropositive potentials to the working electrode under atmospheric pressure (Figure S2). The details of the applied potentials are explained in the comments in Figure S3.

### Preparation of a CoO_*x*_-Loaded BiVO_4_ Photoanode

A CoO_*x*_-loaded BiVO_4_ photoanode was prepared according to a previous report.^54^ Either Bi(NO_3_)_3_·5H_2_O or NH_4_VO_3_ was separately dissolved in an aqueous nitric acid solution (6.5 mol L^–1^) with ultrasonication. Afterward, both the solutions were mixed to obtain a precursor solution. Both of the final concentrations of the aqueous Bi(NO_3_)_3_ and NH_4_VO_3_ solutions were 100 mmol L^–1^. This precursor solution was dripped on an FTO substrate that was cleaned with ozone. The solvent was dried on a hot plate. The amount of the precursor solution dripped on FTO was 5 μL cm^–2^. The FTO substrate coated with the precursor was heated at 773 K for 2 h in air using a muffle furnace to obtain a pristine BiVO_4_ photoanode. One microliter of an aqueous Co(NO_3_)_3_ solution (8 mmol L^–1^) was dripped and spread on the BiVO_4_ photoanode (1 cm^2^). After the solvent dried up at room temperature, the photoanode coated with the cobalt nitrate was heated at 673 K for 1 h to obtain a CoO_*x*_-loaded (Co: 8 nmol cm^–2^) BiVO_4_ photoanode.

### Photoelectrochemical Measurements

Photoelectrochemical measurements were conducted using an H-type glass cell and a potentiostat (Hokuto Denko; HSV-110) (Figure S4). The glass cell was divided into cathode and anode parts by using a Nafion membrane (DuPont). A Pt wire and Ag/AgCl were employed as counter and reference electrodes, respectively. Either an aqueous K_2_SO_4_ solution (0.1 mol L^–1^) with a phosphate buffer (K_2_HPO_4_ and NaH_2_PO_4_, each 0.025 mol L^–1^) or an aqueous KHCO_3_ solution (0.1 mol L^–1^) was employed as an electrolyte. The electrolyte was carefully saturated with either N_2_, Ar, or CO_2_ gas (1 atm) before the photoelectrochemical measurements. A 300 W Xe arc lamp was employed as the light source. The wavelength of the irradiation light was controlled using a cutoff filter (λ > 420 nm), an NIR-absorbing filter, or band-pass filters. The obtained gaseous products were determined using gas chromatographs (Shimadzu GC-8A; TCD, MS-5A, Ar or He carrier, detection for H_2_ or O_2_; FID with a methanizer, MS-13X, N_2_ carrier, detection for CO). An isotope experiment was conducted by using ^13^CO_2_ gas. The ^13^CO of the reduction product was analyzed using GC-MS (Shimadzu; GCMS-QP2010 Plus, RESTEK; RT-Msieve 5A).

### Characterization

A diffuse reflectance spectrum of a CuGaS_2_ powder was recorded using a UV–vis–NIR spectrometer (JASCO; V-570) with an integrating sphere. The reflectance of CuGaS_2_ was converted to absorbance by using the Kubelka–Munk function. Particulate-CuGaS_2_-based photocathodes with and without polymers were characterized using a scanning electron microscope (JEOL; JSM-7600F) and an X-ray photoelectron spectroscope (Shimadzu; ESCA-3400, Mg anode). The deposited polymer was analyzed using Raman (JASCO Corporation, RMP-5300, irradiation light wavelength: 532 nm), the UV–vis–NIR spectrometer in a transmittance measurement mode, X-ray diffraction, and the scanning electron microscope.

## Results and Discussion

### Structural Characterization of a Particulate-CuGaS_2_-Based Photocathode Modified with PPy

**Figure 3:**
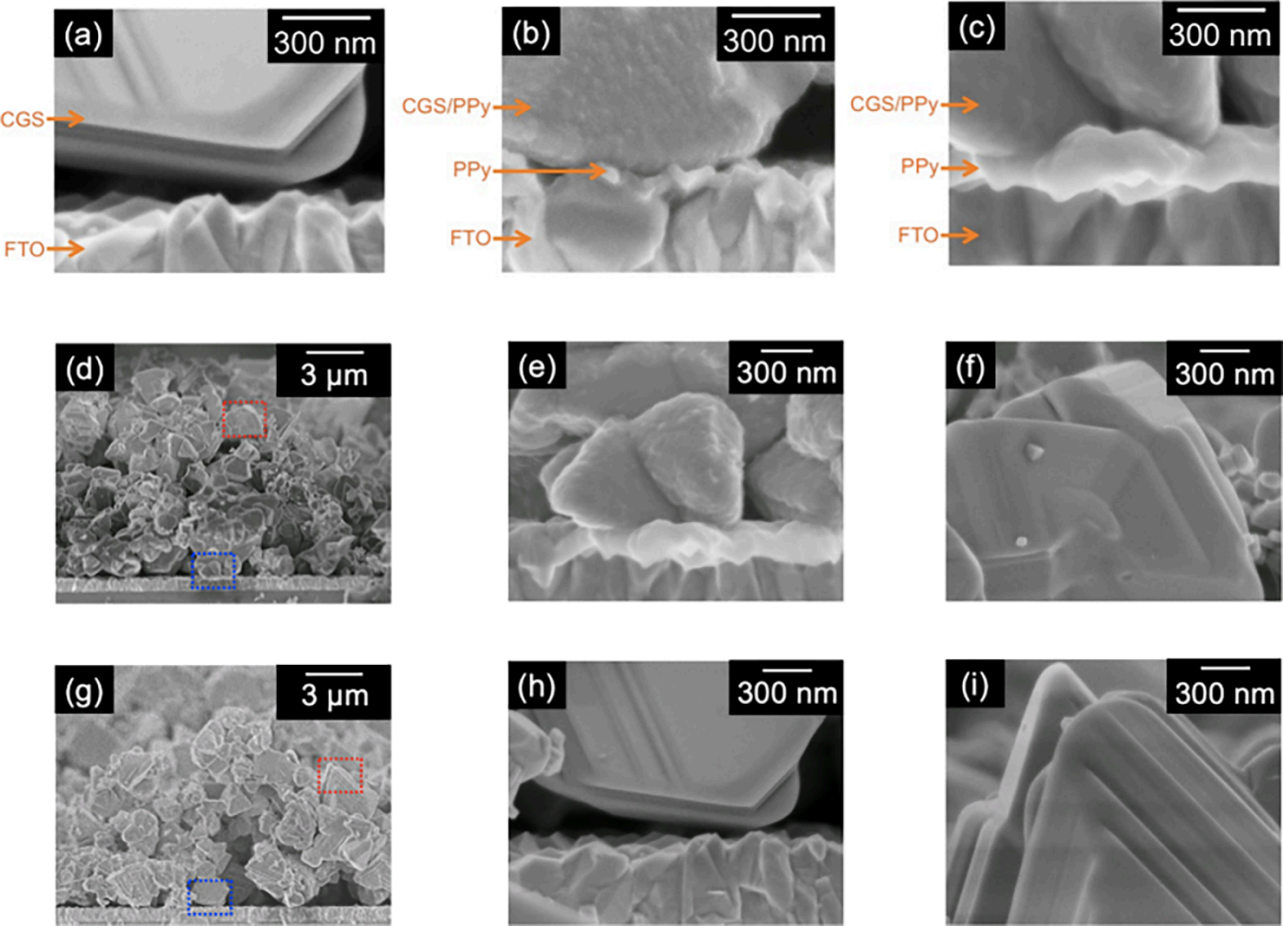
Cross-section SEM images of CuGaS_2_ photocathodes with/without PPy. (a) Bare CuGaS_2_. (b and c) CuGaS_2_ modified with PPy. The electricity was about (b) 20 mC cm^–2^ or (c) 90 mC cm^–2^. Notation of CGS indicates CuGaS_2_. (d) An overview of (c). (e and f) The magnifications of the blue and red dotted squares of (d), respectively. (g) Overview of (a). (h and i) The magnifications of the blue and red dotted squares of (g), respectively. Parts (c) and (e) are almost the same region, and parts (a) and (h) are almost the same region.

**Figure 4:**
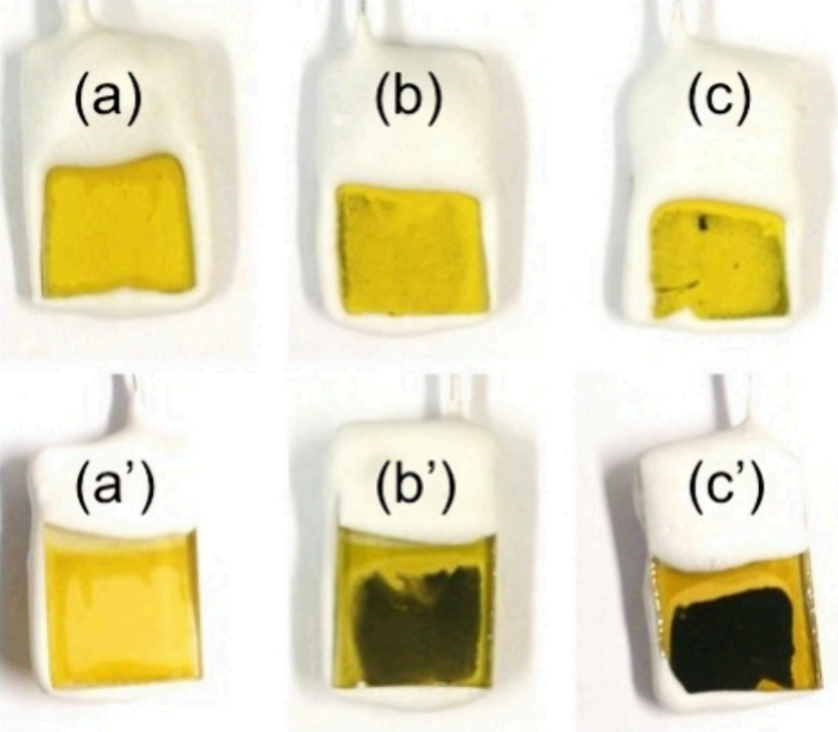
Photographs of the front sides of CuGaS_2_ photocathodes (a) without PPy and (b and c) with PPy. The electricity was about (b) 20 mC cm^–2^ or (c) 90 mC cm^–2^. The notations with prime symbols indicate the corresponding back sides.

A particulate-CuGaS_2_-based photocathode modified with polypyrrole (PPy) was characterized. Note that the amounts of deposited PPy increased with an increase in the amounts of electricity consumed for the formation of PPy (Figures S5–S9), indicating that the amounts of PPy can be controlled by the amounts of the electricity. Figure 3 shows cross-sectional scanning electron microscopy (SEM) images of the CuGaS_2_ photocathodes with/without PPy. A cavity between particulate CuGaS_2_ and an FTO substrate was clearly observed (Figure 3a). PPy was deposited in the cavity (Figure 3b). When the electricity used for an electrochemical oxidative polymerization of PPy increased, the cavity was filled with PPy (Figure 3c). Additionally, the amounts of PPy deposited on the CuGaS_2_ surfaces also increased judging from the disappearance of the angular shapes of the bare particulate CuGaS_2_ (Figures 3a–3c). Thicknesses of the PPy-modified particulate-CuGaS_2_-based photocathode and the bare photocathode were ca. 10 μm in the observed regions (Figures 3d and 3g). By observing the different regions of CuGaS_2_ modified with PPy (Figures 3d–3f), particulate CuGaS_2_ neighboring at the surface of FTO were almost completely wrapped with PPy (Figure 3e), while the particulate CuGaS_2_ far from the FTO was bare (Figure 3f). Particulate CuGaS_2_ without PPy possessed angular shapes regardless of the distance to FTO (Figures 3g–3i). Photographs of the CuGaS_2_ photocathode with/without PPy are shown in Figure 4. The typical yellow color of CuGaS_2_ was observed in front sides of both the samples with/without PPy (Figures 4a–4c). In contrast, in the back sides of those, a typical dark color of PPy with an oxidized state^55^ became deeper with an increase in the electricity (Figures 4b′ and 4c′). Consequentially, it became gradually difficult to recognize the yellow color. In an X-ray photoelectron spectroscopy (XPS) analysis of the N 1s peak corresponding to nitrogen atoms contained in PPy, the peak intensity of PPy-modified CuGaS_2_ was quite small as compared to that of PPy just deposited on FTO (Figure S10). The characterizations of SEM, photographs, and XPS indicated that PPy was deposited mainly in the cavities between particulate CuGaS_2_ and the FTO substrate. The obtained particulate-CuGaS_2_-based photocathodes modified with and without PPy were described as CGS/PPy and CGS, respectively.

### Evaluation of the Photoelectrochemical Property of CGS/PPy for Water and CO_2_ Reduction

**Figure 5:**
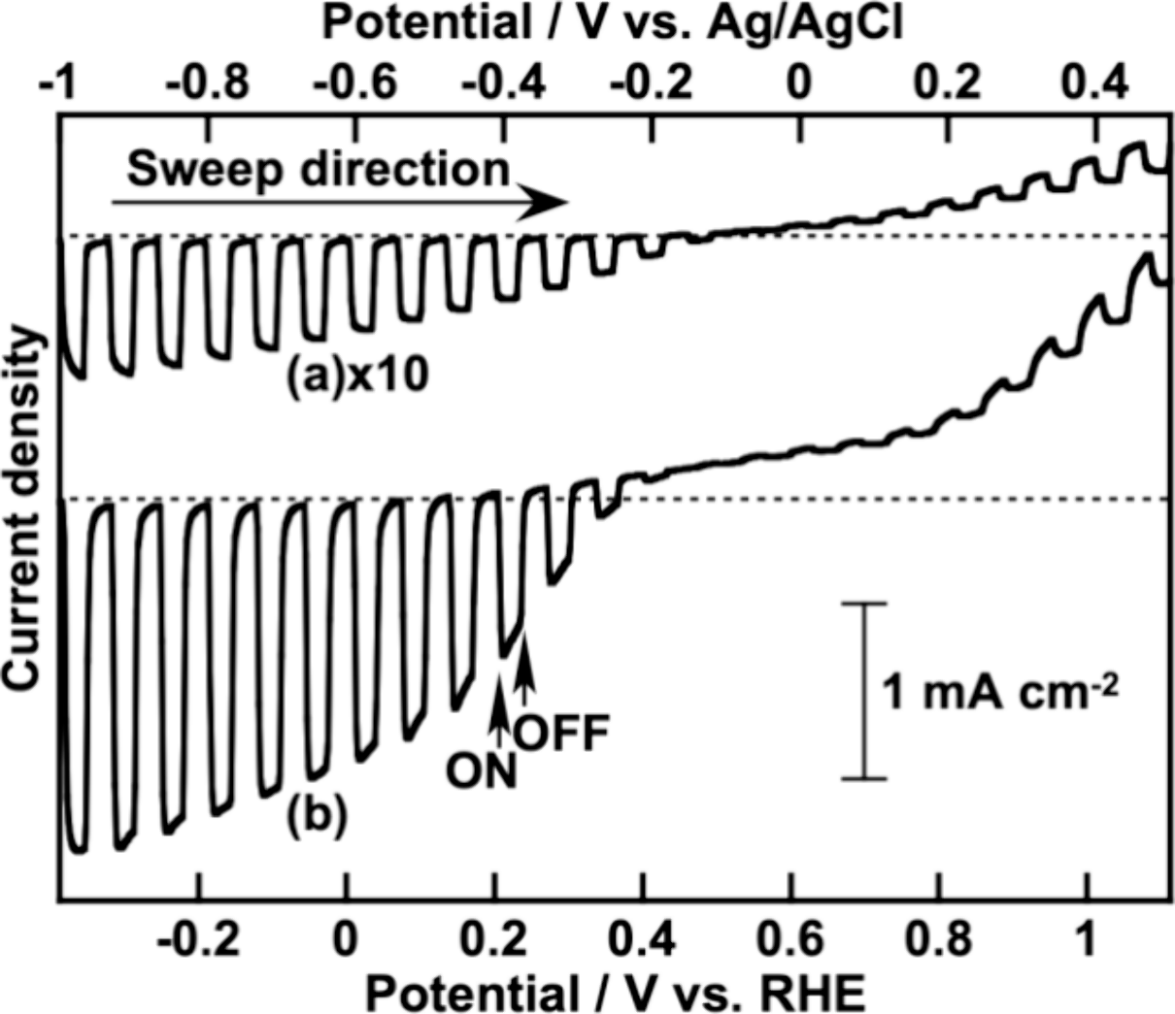
Linear sweep voltammograms under visible light irradiation of (a) a pristine particulate CuGaS_2_ photocathode and (b) a particulate CuGaS_2_ photocathode modified with PPy. Electrolyte, 0.1 mol L^–1^ of K_2_SO_4_ aq. containing a phosphate buffer (pH 7) under 1 atm of N_2_ gas; light source, a 300 W Xe arc lamp with a cutoff filter (λ > 420 nm). The amount of electricity needed to prepare polypyrrole was about 40 mC cm^–2^.

**Figure 6:**
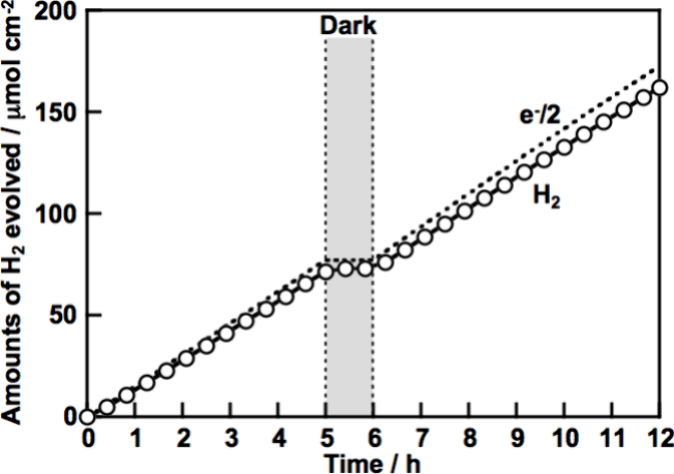
Photoelectrochemical hydrogen evolution under visible light irradiation using a CuGaS_2_ photocathode modified with PPy. Electrolyte, 0.1 mol L^–1^ of K_2_SO_4_ aq. containing a phosphate buffer (pH 7) under 1 atm of Ar gas; light source, a 300 W Xe arc lamp with a cutoff filter (λ > 420 nm); applied potential, 0 V vs RHE (pH 7); area of the photoelectrode, 3.5 cm^2^. The amount of electricity used to prepare polypyrrole was about 50 mC cm^–2^. The amounts of the electrons were also divided by the geometrical area of the electrode (namely, the mole of the amounts of the electrons correspond to the vertical axis scale).

The effects of modification with PPy on the photoelectrochemical property of CGS were investigated. Figure 5 shows the linear sweep voltammograms of CGS with and without PPy under visible light. An aqueous K_2_SO_4_ solution with a phosphate buffer was employed as an electrolyte after it was saturated with 1 atm of N_2_ gas. The cathodic photocurrent density of CGS was drastically improved by PPy modification (Figures 5a and 5b). The anodic dark current of CGS/PPy around the potential from 0 to 0.4 V vs Ag/AgCl was greater than that of CGS. This might be attributed to excessive oxidation of PPy (Figure S11a). Figure 6 shows the time course of H_2_ formation using CGS/PPy under visible light irradiation. The photoelectrochemical reaction to form H_2_ steadily proceeded. The amounts of obtained H_2_ gas agreed well with the half number of electrons passing through the out circuit. This indicated that the observed photocurrent was almost consumed for the reduction of water to form H_2_. Additionally, the cathodic photocurrent of CGS/PPy under simulated solar light was steady for a long time (Figure S12). The improved CGS/PPy was furthermore applied to photoelectrochemical CO_2_ reduction as shown in Table 1. An aqueous KHCO_3_ solution saturated with 1 atm of CO_2_ gas was employed as the electrolyte instead of the aqueous K_2_SO_4_ electrolyte. When unmodified CGS was used, H_2_ and CO were detected (Table 1, entry 1). This is consistent with the CGS thin-film photocathode reported in the literature.^14^ Again, the CGS’s cathodic photocurrent density was also improved by PPy modification accompanied by an increase in the H_2_ and CO formation even under the CO_2_ reduction conditions (Table 1, entry 2). But, Faradaic efficiency for CO formation (FE_CO_) over a PPy-modified CuGaS_2_ photocathode was similar to that over a bare CuGaS_2_ photocathode. This implied that the property of the active sites of the surface of CuGaS_2_ was retained after PPy modification. A cathodic current and gas evolution were negligible in the dark (Table 1, entry 3). CO was not formed over CGS/PPy in an aqueous K_2_SO_4_ solution with a phosphate buffer saturated with 1 atm of N_2_ gas (Table 1, entry 4). ^13^CO was certainly obtained accompanied by no ^12^CO formation in an isotope experiment using ^13^CO_2_ gas (Figure S13). These control experiments revealed that CO was formed not through decomposition of PPy but through reduction of CO_2_. Thus, the role of PPy formed by the electrochemical oxidative polymerization in the cavities between particulate CGS and an FTO substrate was not the active site for CO_2_ reduction but rather a carrier transportation between particulate CGS and an FTO substrate.

**Table 1:** Photoelectrochemical CO_2_ Reduction Using CGS Photocathodes with/without PPy^a^

					amounts of products (μmol) (3 h)			
entry	PPy	gas	light irradiation	current density (mA cm^–2^)	H_2_	CO	*FE*_total_ %^b^	*FE*_CO_ %^c^
1	no	CO_2_	yes	0.006	1.5	0.04	114	3
2	yes	CO_2_	yes	1.7–0.7	32	2	97	6
3	yes	CO_2_	no	trace	trace	0	–	–
4	yes	N_2_	yes	1.6–0.7	52	0	94	0

a Potential, –0.6 V vs Ag/AgCl; electrolyte, 0.1 mol L^–1^ KHCO_3_ aq. saturated with 1 atm of CO_2_ gas (almost neutral pH) or 0.1 mol L^–1^ K_2_SO_4_ aq. containing a phosphate buffer saturated with 1 atm of N_2_ gas (pH 7); light source, a 300 W Xe arc lamp with a cutoff filter (λ > 420 nm). The amounts of electricity to prepare polypyrroles were about 50 mC cm^–2^. Current density means the maximum (early period) and minimum (later period) values recorded.

b *FE*_total_ = (sum of electron numbers consumed for H_2_ and CO formations)/(sum of electron numbers electrons passing through the out circuit) × 100.

c *FE*_CO_ = (sum of electron numbers consumed for CO formation)/(sum of electron numbers electrons passing through the out circuit) × 100.

**Figure 7:**
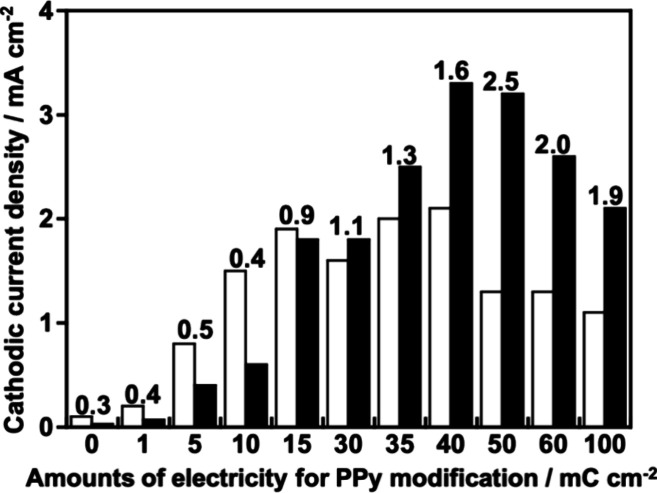
Dependence of the amounts of modified PPy on the photocurrent density of the CuGaS_2_ photocathode. Values of *R*_front/back_ = (*C*_front_)/(*C*_back_) were displayed on the respective tops of the bar graphs. *C*_front_ corresponds to the black bar lengths, and *C*_back_ corresponds to the white bar lengths. Potential, 0 V vs RHE; electrolyte, 0.1 mol L^–1^ K_2_SO_4_ aq. containing a phosphate buffer (pH 7) under 1 atm of N_2_ gas; light source, a 300 W Xe arc lamp with a cutoff filter and an NIR-absorbing filter (λ > 420 nm).

The evidence that PPy worked as a carrier transporter was experimentally confirmed by the dependence of the cathodic photocurrent densities of CGS upon the amounts of deposited PPy, as shown in Figure 7. An aqueous K_2_SO_4_ solution with a phosphate buffer was employed as an electrolyte after it was saturated with 1 atm of N_2_ gas, and irradiation light was visible. CGS/PPy photocathodes were irradiated from the particulate CGS side (front side) and the FTO substrate side (back side). A ratio of the front sides’ currents (*C*_front_) and back sides’ currents (*C*_back_) was described as *R*_front/back_, namely, *R*_front/back_ = (*C*_front_)/(*C*_back_). An increase in the value of the horizontal axis in Figure 7 indicates an increase in the amounts of deposited PPy. Note that PPy-deposited (50 mC cm^–2^) FTO without CGS gave a negligible cathodic photocurrent smaller than 1 μA cm^–2^ in the same condition as Figure 7. *R*_front/back_ was beyond 1 when the amounts of electricity were larger than 30 mC cm^–2^. This can be interpreted as follows. At first, as discussed in the literature about an RGO-composited CGS system,^10^ most of the holes photogenerated in bare particulate CGS cannot arrive at an FTO substrate. This is due to a number of cavities between particulate CGS and an FTO substrate. In other words, most of the particulate CGS are far from FTO, resulting in a small amount of CGS neighbors on FTO. Therefore, in general, cathodic photocurrents of bare particulate CGS obtained with irradiation to a front side tend to be low as compared to the back side’s current. Contrary, considering our characterization shown in Figures 3, 4, and S10, PPy in the cavities connected most of particulate CGS to an FTO substrate. This would lead to better carrier transportation as proposed in Figure 1, resulting in an increase in the photocurrents (Figure 7). When the amounts of electricity were larger than 40 mC cm^–2^, both *C*_front_ (black bars) and *C*_back_ (white bars) decreased due to shielding of the irradiation light by deposited PPy with dark color. Thus, PPy has arisen as an efficient carrier transporter between particulate CGS to an FTO substrate.

### Effects of Polymers’ Redox Potentials on CGS’s Photoelectrochemical Performances

**Figure 8:**
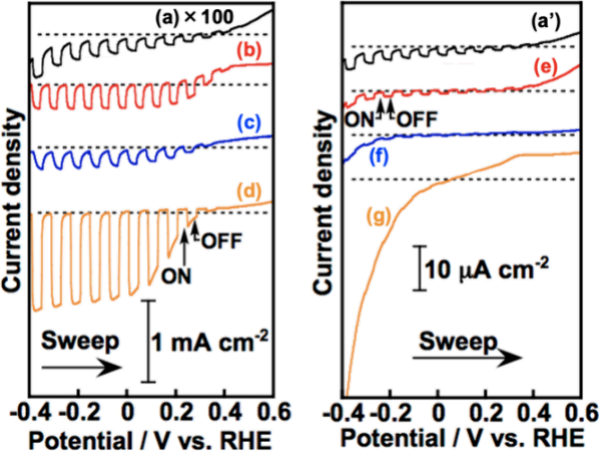
Linear sweep voltammograms of particulate-CuGaS_2_-based photocathodes modified with (a and a′) no polymer, (b) PPy, (c) PEDOP, (d) PEDOT, (e) PT, (f) PMP3C, and (g) P3HT under simulated sunlight irradiation. Electrolyte, 0.1 mol L^–1^ of K_2_SO_4_ aq. containing a phosphate buffer (pH 7) under 1 atm of Ar gas; light source, a solar simulator (AM-1.5G). The amounts of electricity for the electrochemical oxidative polymerization of PPy, PEDOP, PEDOT, PT, PMP3C, and P3HT were almost 80, 80, 75, 280, 95, and 330, respectively. Potential sweep rate was 10 mV s^–1^.

**Figure 9:**
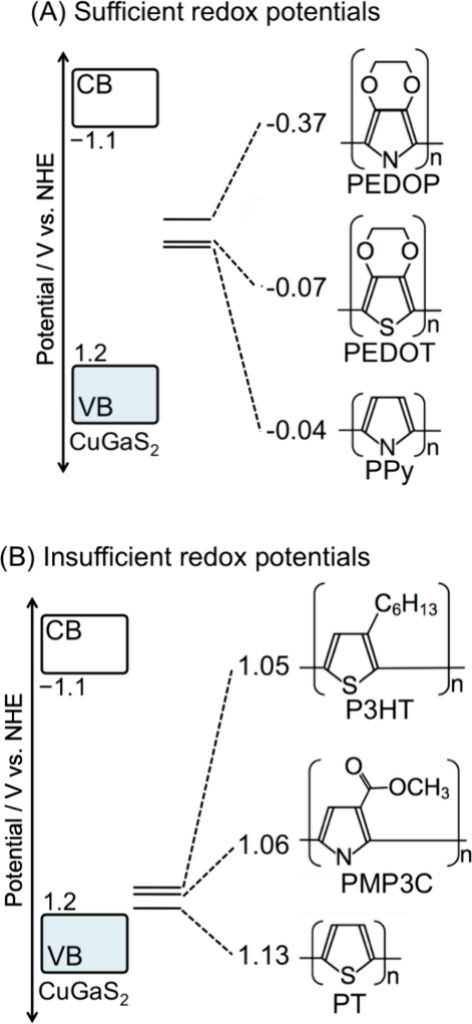
Proposed band diagrams of powdered CuGaS_2_ material and organic conductive polymers. CB and VB mean conduction band and valence band, respectively. The horizontal solid lines indicate the redox potentials of the polymers.

To further clarify the mechanism of the carrier migration between particulate CGS and an FTO substrate, the effects of redox potentials of conductive organic polymers on the CGS’s photocurrents were evaluated, as shown in Figure 8. Structures of the employed polymers are summarized in Figure 2. An aqueous K_2_SO_4_ solution with a phosphate buffer saturated with 1 atm of Ar gas was used in addition to a solar simulator as the light source. PPy, PEDOP, and PEDOT improved the cathodic photocurrents. In contrast, PT, PMP3C, and P3HT made the photocurrents lower than that of bare CGS. Onsets of the photocurrents were slightly shifted to negative positions by modification with PPy, PEDOT, and PEDOP as compared to that of a bare CGS. The onsets of CGS modified with PT, PMP3C, and P3HT were unclear. Among them, PEDOT was the most effective in improving the photocurrent. Figure 9 shows an energy diagram of the polymers’ redox potentials that were estimated by cyclic voltammetry (Figure S11) in addition to a band position of CuGaS_2_ reported in the literature.^56^ The polymers were classified into two groups. One is group A, in which the redox potentials are significantly negative compared with the valence band maximum (VBM) of CGS. The second group B is opposite group A. Group A gave a significant effect on the cathodic photocurrent of CGS because of the large potential difference between the redox potential and the VBM, enhancing the transportation of the hole from metal sulfide to an FTO substrate.

An action spectrum of CGS/PEDOT was measured. The photon number of the monochromatic irradiation light (Asahi Spectra; MAX-301 with band-pass filters) was measured by using a photodiode head (OPHIRA; PD300-UV) and a power monitor (NOVA). The IPCEs were calculated as follows.
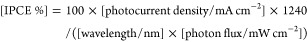


**Figure 10:**
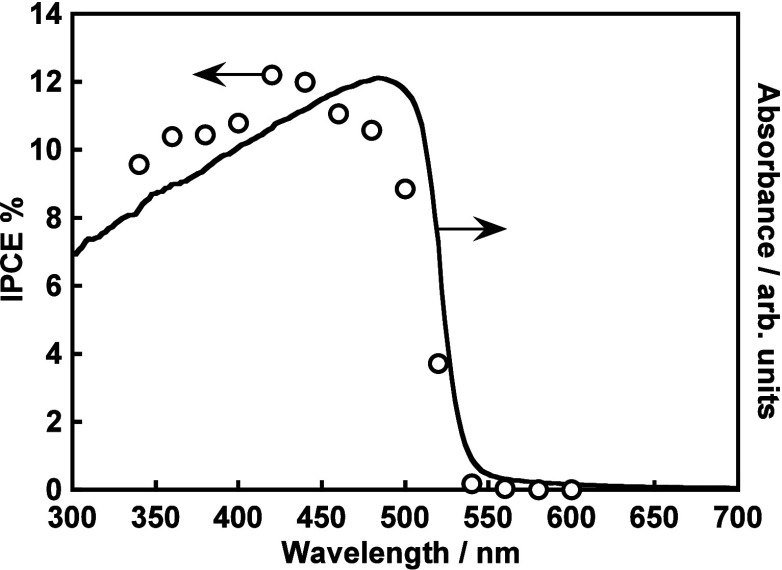
Diffuse reflectance spectrum of a pristine particulate CuGaS_2_ and IPCEs for hydrogen evolution using a particulate CuGaS_2_ photocathode modified with PEDOT. Electrolyte, 0.1 mol L^–1^ of K_2_SO_4_ aq. containing a phosphate buffer under 1 atm of N_2_ gas; light source, a 300 W Xe arc lamp with band-path filters; potential, 0 V vs RHE (pH 7).

An onset of the action spectrum agreed well with an absorption edge of the CGS powder, as shown in Figure 10. It is noteworthy that the IPCE of CGS/PEDOT corresponding to water reduction to form H_2_ (ca. 11%@450 nm, −0.6 V vs Ag/AgCl) was close to an IPCE of a CGS thin-film photocathode prepared by a vacuum process for reduction of Eu^3+^ cations (ca. 15%@450 nm, −0.6 V vs Ag/AgCl).^14^ The photoresponse of the CGS/PEDOT was not due to PEDOT because the onset of the action spectrum did not agree with an absorption spectrum of PEDOT.^57^ For another insight of hole transportation in the interface, it is reported that a detrimental Schottky junction forms at the interface between *p*-type chalcogenides and *n*-FTO due to the work function difference of the two materials.^58^ This hinders hole transfer to FTO. The present conducting polymers may remove this junction, allowing hole transfer without a barrier, similar to the effect of a carbon layer in the particle-transfer method.^25^ Thus, it is concluded that PEDOT modification has arisen as an efficient methodology to improve the photoelectrochemical performance of particulate CuGaS_2_ without any vacuum processes in the modification. The PEDOT efficiently worked as a solid-state hole transporter from particulate CuGaS_2_ to an FTO substrate, as proposed in Figure 1. This mechanism is different from the related other applications of the conductive polymers, such as accelerating the consumption of hole scavengers over quantum dots,^59^ harvesting photon energy for photoelectrochemical hydrogen production,^60,61^ improving consumption of electrons for photoelectrochemical hydrogen production,^62^ and boosting the surface reaction for CO_2_ reduction by *p*-ZnTe.^63^

### Artificial Photosynthesis upon Combining a CGS/PEDOT Photocathode with a CoO_*x*_/BiVO_4_ Photoanode

**Figure 11:**
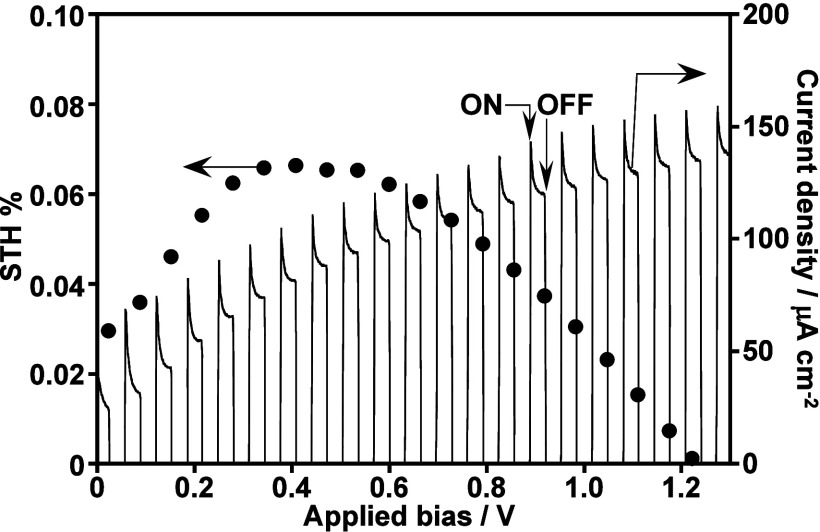
Solar energy conversion efficiency (left axis) and current (right axis) obtained using a photoelectrochemical cell consisting of a CuGaS_2_/PEDOT photocathode (3.8 cm^2^) and a CoO_*x*_-loaded BiVO_4_ thin-film photoanode (0.9 cm^2^). Electrolyte, 0.1 mol L^–1^ of K_2_SO_4_ aq. containing a phosphate buffer (pH 7) saturated with Ar gas (1 atm); light source, a solar simulator (AM-1.5G).

**Figure 12:**
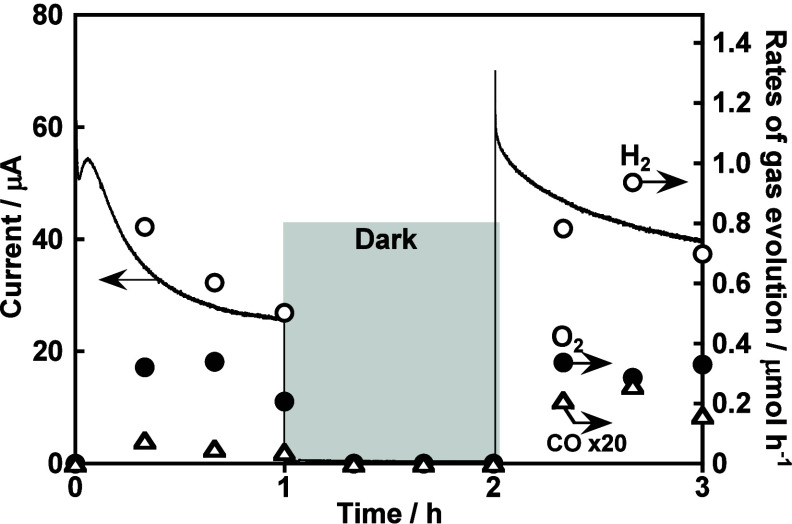
CO_2_ reduction utilizing water as an electron donor using a photoelectrochemical cell consisting of a CuGaS_2_/PEDOT photocathode (3 cm^2^) and a CoO_*x*_-loaded BiVO_4_ thin-film photoanode (1 cm^2^). Electrolyte, 0.1 mol L^–1^ of KHCO_3_ aq. saturated with CO_2_ gas (1 atm) at about pH 7; light source, a solar simulator (AM-1.5G); applied bias, 0.4 V.

CGS/PEDOT was applied to a photoelectrochemical cell for solar water splitting upon combining with a CoO_*x*_/BiVO_4_ photoanode, as shown in Figure 11. The cell was a typical H-type one divided into cathode and anode parts by a Nafion membrane. An aqueous K_2_SO_4_ solution with a phosphate buffer saturated with 1 atm of Ar gas was used in addition to a solar simulator as the light source. The photocurrent was observed even without an external bias due to an overlap of onsets of a CGS/PEDOT photocathode and a CoO_*x*_/BiVO_4_ photoanode (Figure S14). The maximum efficiency for solar water splitting using the photoelectrochemical cell achieved 0.065% around 0.4 V of applied bias, which was estimated by the following equation:
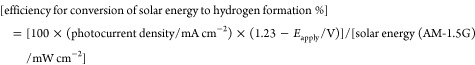
where *E*_apply_ indicates applied bias between a photocathode and a photoanode. Furthermore, the photoelectrochemical cell was applied to an artificial photosynthetic CO_2_ reduction, as shown in Figure 12. An aqueous KHCO_3_ solution saturated with 1 atm of CO_2_ gas was employed as the electrolyte instead of the K_2_SO_4_ electrolyte. H_2_ and CO were obtained as the reduction products of H_2_O and CO_2_ separately from O_2_ formation ascribing to an oxidation product of H_2_O. When the K_2_SO_4_ electrolyte saturated with Ar was used, CO was not detected. These indicated that water was certainly consumed as the electron source for syngas (H_2_ and CO mixture gas) formation through the reduction of water and CO_2_. Thus, it was demonstrated that a particulate-CuGaS_2_-based photocathode modified with PEDOT was useful as a building block for photoelectrochemical cells to achieve water splitting and the CO_2_ reduction of artificial photosynthesis.

## Conclusions

We discovered a methodology to make PPy a carrier transporter in a cathodic photoelectrochemical reaction. The electrochemical oxidative polymerization made the cavities between particulate CuGaS_2_ photocatalysts and an FTO substrate fill with PPy, which was a crucial factor in enhancing hole transportation. Moreover, the same approach was effective for PEDOT and PEDOP modification. The mechanism of the carrier transportation using PPy, PEDOT, and PEDOP was ascribed to the promotion of hole migration from particulate CuGaS_2_ photocatalysts to an FTO substrate. Furthermore, the PEDOT-modified CuGaS_2_ photocathode was useful for constructing a photoelectrochemical cell to achieve artificial photosynthesis of water splitting and CO_2_ reduction under simulated solar light upon combining with a CoO_*x*_/BiVO_4_ photoanode. Most importantly, this modification can be performed not under vacuum conditions but under ambient pressure. The knowledge in this work is strongly expected to contribute to spreading out employing particulate photocatalysts possessing *p*-type semiconductor character toward making highly efficient photocathodes for water and CO_2_ reduction under sunlight with a large-scalable process.

## Supporting Information Available

The experiment setups and the characterization for the photocatalyst and the polymers (linear sweep voltammograms, chronoamperometry, isotope experiment, photographs, UV–vis, XRD, Raman, SEM, and XPS) (PDF)

## References

[ref1] Walter M. G.; Warren E. L.; McKone J. R.; Boettcher S. W.; Mi Q.; Santori E. A.; Lewis N. S. Solar Water Splitting Cells. Chem. Rev. 2010, 110, 6446–6473. 10.1021/cr1002326. 21062097

[ref2] Hisatomi T.; Kubota J.; Domen K. Recent advances in semiconductors for photocatalytic and photoelectrochemical water splitting. Chem. Soc. Rev. 2014, 43, 7520–7535. 10.1039/C3CS60378D. 24413305

[ref3] Dey A.; Maiti D.; Lahiri G. K. Photoelectrocatalytic Reduction of CO_2_ into C1 Products by Using Modified-Semiconductor-Based Catalyst Systems. Asian J. Org. Chem. 2017, 6, 1519–1530. 10.1002/ajoc.201700351.

[ref4] Kamiya K.; Fujii K.; Sugiyama M.; Nakanishi S. CO_2_ Electrolysis in Integrated Artificial Photosynthesis Systems. Chem. Lett. 2021, 50, 166–179. 10.1246/cl.200691.

[ref5] Yoshino S.; Takayama T.; Yamaguchi Y.; Iwase A.; Kudo A. CO_2_ Reduction Using Water as an Electron Donor over Heterogeneous Photocatalysts Aiming at Artificial Photosynthesis. Acc. Chem. Res. 2022, 55, 966–977. 10.1021/acs.accounts.1c00676. 35230087 PMC8988292

[ref6] van Embden J.; Latham K.; Duffy N. W.; Tachibana Y. Near-Infrared Absorbing Cu_12_Sb_4_S_13_ and Cu_3_SbS_4_ Nanocrystals: Synthesis, Characterization, and Photoelectrochemistry. J. Am. Chem. Soc. 2013, 135, 11562–11571. 10.1021/ja402702x. 23876109

[ref7] Septina W.; Gunawan; Ikeda S.; Harada T.; Higashi M.; Abe R.; Matsumura M. Photosplitting of Water from Wide-Gap Cu(In,Ga)S_2_ Thin Films Modified with a CdS Layer and Pt Nanoparticles for a High-Onset-Potential Photocathode. J. Phys. Chem. C 2015, 119, 8576–8583. 10.1021/acs.jpcc.5b02068.

[ref8] Kato T.; Hakari Y.; Ikeda S.; Jia Q.; Iwase A.; Kudo A. Utilization of Metal Sulfide Material of (CuGa)_1-x_Zn_2x_S_2_ Solid Solution with Visible Light Response in Photocatalytic and Photoelectrochemical Solar Water Splitting Systems. J. Phys. Chem. Lett. 2015, 6, 1042–1047. 10.1021/acs.jpclett.5b00137. 26262867

[ref9] Kaga H.; Tsutsui Y.; Nagane A.; Iwase A.; Kudo A. An effect of Ag(I)-substitution at Cu sites in CuGaS_2_ on photocatalytic and photoelectrochemical properties for solar hydrogen evolution. J. Mater. Chem. A 2015, 3, 21815–21823. 10.1039/C5TA04756K.

[ref10] Iwase A.; Ng Y. H.; Amal R.; Kudo A. Solar hydrogen evolution using a CuGaS_2_ photocathode improved by incorporating reduced graphene oxide. J. Mater. Chem. A 2015, 3, 8566–8570. 10.1039/C5TA01237F.

[ref11] Wu Y.; Yue Z.; Liu A.; Yang P.; Zhu M. P-Type Cu-Doped Zn_0.3_Cd_0.7_S/Graphene Photocathode for Efficient Water Splitting in a Photoelectrochemical Tandem Cell. ACS Sustainable Chem. Eng. 2016, 4, 2569–2577. 10.1021/acssuschemeng.5b01795.

[ref12] Kamimura S.; Sasaki Y.; Kanaya M.; Tsubota T.; Ohno T. Improvement of selectivity for CO_2_ reduction by using Cu_2_ZnSnS_4_ electrodes modified with different buffer layers (CdS and In_2_S_3_) under visible light irradiation. RSC Adv. 2016, 6, 112594–112601. 10.1039/C6RA22546B.

[ref13] Hayashi T.; Niishiro R.; Ishihara H.; Yamaguchi M.; Jia Q.; Kuang Y.; Higashi T.; Iwase A.; Minegishi T.; Yamada T.; Domen K.; Kudo A. Powder-based (CuGa_1-y_In_y_)_1-x_Zn_2x_S_2_ solid solution photocathodes with a largely positive onset potential for solar water splitting. Sustainable Energy Fuels 2018, 2, 2016–2024. 10.1039/C8SE00079D.

[ref14] Ikeda S.; Tanaka Y.; Kawaguchi T.; Fujikawa S.; Harada T.; Takayama T.; Iwase A.; Kudo A. Photoelectrochemical Reduction of CO_2_ to CO Using a CuGaS_2_ Thin-film Photocathode Prepared by a Spray Pyrolysis Method. Chem. Lett. 2018, 47, 1424–1427. 10.1246/cl.180720.

[ref15] Ikeda S.; Aono N.; Iwase A.; Kobayashi H.; Kudo A. Cu_3_MS_4_ (M = V, Nb, Ta) and its Solid Solutions with Sulvanite Structure for Photocatalytic and Photoelectrochemical H_2_ Evolution under Visible-Light Irradiation. ChemSusChem 2019, 12, 1977–1983. 10.1002/cssc.201802702. 30666792

[ref16] Gaillard N.; Prasher D.; Chong M.; Deangelis A.; Horsley K.; Ishii H. A.; Bradley J. P.; Varley J.; Ogitsu T. Wide-Bandgap Cu(In,Ga)S_2_ Photocathodes Integrated on Transparent Conductive F:SnO_2_ Substrates for Chalcopyrite-Based Water Splitting Tandem Devices. ACS Appl. Energy Mater. 2019, 2, 5515–5524. 10.1021/acsaem.9b00690.

[ref17] Ikeda S.; Fujikawa S.; Harada T.; Nguyen T. H.; Nakanishi S.; Takayama T.; Iwase A.; Kudo A. Photocathode Characteristics of a Spray-Deposited Cu_2_ZnGeS_4_ Thin Film for CO_2_ Reduction in a CO_2_-Saturated Aqueous Solution. ACS Appl. Energy Mater. 2019, 2, 6911–6918. 10.1021/acsaem.9b01418.

[ref18] Yoshino S.; Iwase A.; Ng Y. H.; Amal R.; Kudo A. Z-Schematic Solar Water Splitting Using Fine Particles of H_2_-Evolving (CuGa)_0.5_ZnS_2_ Photocatalyst Prepared by a Flux Method with Chloride Salts. ACS Appl. Energy Mater. 2020, 3, 5684–5692. 10.1021/acsaem.0c00661.

[ref19] Kageshima Y.; Shiga S.; Ode T.; Takagi F.; Shiiba H.; Htay M. T.; Hashimoto Y.; Teshima K.; Domen K.; Nishikiori H. Photocatalytic and Photoelectrochemical Hydrogen Evolution from Water over Cu_2_Sn_x_Ge_1-x_S_3_ Particles. J. Am. Chem. Soc. 2021, 143, 5698–5708. 10.1021/jacs.0c12140. 33827207

[ref20] Wu L.; Su F.; Liu T.; Liu G.-Q.; Li Y.; Ma T.; Wang Y.; Zhang C.; Yang Y.; Yu S.-H. Phosphorus-Doped Single-Crystalline Quaternary Sulfide Nanobelts Enable Efficient Visible-Light Photocatalytic Hydrogen Evolution. J. Am. Chem. Soc. 2022, 144, 20620–20629. 10.1021/jacs.2c07313. 36332107

[ref21] Kumagai H.; Minegishi T.; Moriya Y.; Kubota J.; Domen K. Photoelectrochemical Hydrogen Evolution from Water Using Copper Gallium Selenide Electrodes Prepared by a Particle Transfer Method. J. Phys. Chem. C 2014, 118, 16386–16392. 10.1021/jp409921f.

[ref22] Zhang L.; Minegishi T.; Kubota J.; Domen K. Hydrogen evolution from water using Ag_x_Cu_1-x_GaSe_2_ photocathodes under visible light. Phys. Chem. Chem. Phys. 2014, 16, 6167–6174. 10.1039/c3cp54590c. 24562096

[ref23] Chae S. Y.; Park S. J.; Han S. G.; Jung H.; Kim C.-W.; Jeong C.; Joo O.-S.; Min B. K.; Hwang Y. J. Enhanced Photocurrents with ZnS Passivated Cu(In,Ga)(Se,S)_2_ Photocathodes Synthesized Using a Nonvacuum Process for Solar Water Splitting. J. Am. Chem. Soc. 2016, 138, 15673–15681. 10.1021/jacs.6b09595. 27934030

[ref24] Goto Y.; Minegishi T.; Kageshima Y.; Higashi T.; Kaneko H.; Kuang Y.; Nakabayashi M.; Shibata N.; Ishihara H.; Hayashi T.; Kudo A.; Yamada T.; Domen K. A particulate (ZnSe)_0.85_(CuIn_0.7_Ga_0.3_Se_2_)_0.15_ photocathode modified with CdS and ZnS for sunlight-driven overall water splitting. J. Mater. Chem. A 2017, 5, 21242–21248. 10.1039/C7TA06663E.

[ref25] Kageshima Y.; Minegishi T.; Goto Y.; Kaneko H.; Domen K. Particulate photocathode composed of (ZnSe)_0.85_(CuIn_0.7_Ga_0.3_Se_2_)_0.15_ synthesized with Na_2_S for enhanced sunlight-driven hydrogen evolution. Sustainable Energy Fuels 2018, 2, 1957–1965. 10.1039/C8SE00101D.

[ref26] Frick J. J.; Cava R. J.; Bocarsly A. B. Chalcopyrite CuIn(S_1-x_Se_x_)_2_ for Photoelectrocatalytic H_2_ Evolution: Unraveling the Energetics and Complex Kinetics of Photogenerated Charge Transfer in the Semiconductor Bulk. Chem. Mater. 2018, 30, 4422–4431. 10.1021/acs.chemmater.8b01827.

[ref27] Kim B.; Park G.-S.; Chae S. Y.; Kim M. K.; Oh H.-S.; Hwang Y. J.; Kim W.; Min B. K. A highly efficient Cu(In,Ga)(S,Se)_2_ photocathode without a hetero-materials overlayer for solar-hydrogen production. Sci. Rep. 2018, 8, 5182 10.1038/s41598-018-22827-3. 29581436 PMC5980086

[ref28] Hellstern T. R.; Palm D. W.; Carter J.; DeAngelis A. D.; Horsley K.; Weinhardt L.; Yang W.; Blum M.; Gaillard N.; Heske C.; Jaramillo T. F. Molybdenum Disulfide Catalytic Coatings via Atomic Layer Deposition for Solar Hydrogen Production from Copper Gallium Diselenide Photocathodes. ACS Appl. Energy Mater. 2019, 2, 1060–1066. 10.1021/acsaem.8b01562.

[ref29] Kim B.; Park G. S.; Hwang Y. J.; Won D. H.; Kim W.; Lee D. K.; Min B. K. Cu(In,Ga)(S,Se)_2_ Photocathodes with a Grown-In Cu_x_S Catalyst for Solar Water Splitting. ACS Energy Lett. 2019, 4, 2937–2944. 10.1021/acsenergylett.9b01816.

[ref30] Minegishi T.; Yamaguchi S.; Sugiyama M. Efficient hydrogen evolution from water over thin film photocathode composed of solid solutions between ZnSe and Cu(In, Ga)Se_2_ with composition gradient. Appl. Phys. Lett. 2021, 119, 123905 10.1063/5.0064658.

[ref31] Li Z.; Zhong W.; Gao D.; Chen F.; Yu H. CdIn_2_S_4-x_Se_x_ Solid-Solution Nanocrystal Photocatalyst: One-Step Hydrothermal Synthesis, Controllable Band Structure, and Improved H_2_-Evolution Activity. Adv. Sustainable Syst. 2023, 7, 2200030 10.1002/adsu.202200030.

[ref32] Sullivan I.; Zoellner B.; Maggard P. A. Copper(I)-Based p-Type Oxides for Photoelectrochemical and Photovoltaic Solar Energy Conversion. Chem. Mater. 2016, 28, 5999–6016. 10.1021/acs.chemmater.6b00926.

[ref33] Iwashina K.; Iwase A.; Nozawa S.; Adachi S.; Kudo A. Visible-Light-Responsive CuLi_1/3_Ti_2/3_O_2_ Powders Prepared by a Molten CuCl Treatment of Li_2_TiO_3_ for Photocatalytic H_2_ Evolution and Z-Schematic Water Splitting. Chem. Mater. 2016, 28, 4677–4685. 10.1021/acs.chemmater.6b01557.

[ref34] Zoellner B.; Stuart S.; Chung C.-C.; Dougherty D. B.; Jones J.; Maggard P. A. CuNb_1-x_Ta_x_O_3_ (*x* ≤ 0.25) solid solutions: impact of Ta(V) substitution and Cu(I) deficiency on their structure, photocatalytic, and photoelectrochemical properties. J. Mater. Chem. A 2016, 4, 3115–3126. 10.1039/C5TA06609C.

[ref35] Liu C.; Chang Y.-H.; Chen J.; Feng S.-P. Electrochemical Synthesis of Cu_2_O Concave Octahedrons with High-Index Facets and Enhanced Photoelectrochemical Activity. ACS Appl. Mater. Interfaces 2017, 9, 39027–39033. 10.1021/acsami.7b12076. 29039198

[ref36] Tsuji I.; Kato H.; Kudo A. Visible-Light-Induced H_2_ Evolution from an Aqueous Solution Containing Sulfide and Sulfite over a ZnS-CuInS_2_-AgInS_2_ Solid-Solution Photocatalyst. Angew. Chem., Int. Ed. 2005, 44, 3565–3568. 10.1002/anie.200500314. 15880535

[ref37] Tsuji I.; Kato H.; Kobayashi H.; Kudo A. Photocatalytic H_2_ Evolution under Visible-Light Irradiation over Band-Structure-Controlled (CuIn)_x_Zn_2(1-x)_S_2_ Solid Solutions. J. Phys. Chem. B 2005, 109, 7323–7329. 10.1021/jp044722e. 16851838

[ref38] Tsuji I.; Kato H.; Kudo A. Photocatalytic Hydrogen Evolution on ZnS-CuInS_2_-AgInS_2_ Solid Solution Photocatalysts with Wide Visible Light Absorption Bands. Chem. Mater. 2006, 18, 1969–1975. 10.1021/cm0527017.

[ref39] Tsuji I.; Shimodaira Y.; Kato H.; Kobayashi H.; Kudo A. Novel Stannite-type Complex Sulfide Photocatalysts A^I^_2_-Zn-A^IV^-S_4_ (A^I^ = Cu and Ag; A^IV^ = Sn and Ge) for Hydrogen Evolution under Visible-Light Irradiation. Chem. Mater. 2010, 22, 1402–1409. 10.1021/cm9022024.

[ref40] Yang J.; Fu H.; Yang D.; Gao W.; Cong R.; Yang T. ZnGa_2-x_In_x_S_4_ (0 ≤ *x* ≤ 0.4) and Zn_1–2y_(CuGa)_y_Ga_1.7_In_0.3_S_4_ (0.1 ≤ *y* ≤ 0.2): Optimize Visible Light Photocatalytic H_2_ Evolution by Fine Modulation of Band Structures. Inorg. Chem. 2015, 54, 2467–2473. 10.1021/ic503101s. 25695506

[ref41] Quintans C. S.; Kato H.; Kobayashi M.; Kaga H.; Iwase A.; Kudo A.; Kakihana M. Improvement of hydrogen evolution under visible light over Zn_1–2x_(CuGa)_x_Ga_2_S_4_ photocatalysts by synthesis utilizing a polymerizable complex method. J. Mater. Chem. A 2015, 3, 14239–14244. 10.1039/C5TA02114F.

[ref42] Takayama T.; Tsuji I.; Aono N.; Harada M.; Okuda T.; Iwase A.; Kato H.; Kudo A. Development of Various Metal Sulfide Photocatalysts Consisting of d^0^, d^5^, and d^10^ Metal Ions for Sacrificial H_2_ Evolution under Visible Light Irradiation. Chem. Lett. 2017, 46, 616–619. 10.1246/cl.161192.

[ref43] Yoshino S.; Iwase A.; Yamaguchi Y.; Suzuki T. M.; Morikawa T.; Kudo A. Photocatalytic CO_2_ Reduction Using Water as an Electron Donor under Visible Light Irradiation by Z-Scheme and Photoelectrochemical Systems over (CuGa)_0.5_ZnS_2_ in the Presence of Basic Additives. J. Am. Chem. Soc. 2022, 144, 2323–2332. 10.1021/jacs.1c12636. 35076230 PMC8832390

[ref44] Iwashina K.; Iwase A.; Ng Y. H.; Amal R.; Kudo A. Z-Schematic Water Splitting into H_2_ and O_2_ Using Metal Sulfide as a Hydrogen-Evolving Photocatalyst and Reduced Graphene Oxide as a Solid-State Electron Mediator. J. Am. Chem. Soc. 2015, 137, 604–607. 10.1021/ja511615s. 25551584

[ref45] Iwase A.; Yoshino S.; Takayama T.; Ng Y. H.; Amal R.; Kudo A. Water Splitting and CO_2_ Reduction under Visible Light Irradiation Using Z-Scheme Systems Consisting of Metal Sulfides, CoO_x_-Loaded BiVO_4_, and a Reduced Graphene Oxide Electron Mediator. J. Am. Chem. Soc. 2016, 138, 10260–10264. 10.1021/jacs.6b05304. 27459021

[ref46] Pan Z.; Hisatomi T.; Wang Q.; Chen S.; Iwase A.; Nakabayashi M.; Shibata N.; Takata T.; Katayama M.; Minegishi T.; Kudo A.; Domen K. Photoreduced Graphene Oxide as a Conductive Binder to Improve the Water Splitting Activity of Photocatalyst Sheets. Adv. Funct. Mater. 2016, 26, 7011–7019. 10.1002/adfm.201602657.

[ref47] Takayama T.; Sato K.; Fujimura T.; Kojima Y.; Iwase A.; Kudo A. Photocatalytic CO_2_ reduction using water as an electron donor by a powdered Z-scheme system consisting of metal sulfide and an RGO-TiO_2_ composite. Faraday Discuss. 2017, 198, 397–407. 10.1039/C6FD00215C. 28287650

[ref48] Wang Q.; Hisatomi T.; Jia Q.; Tokudome H.; Zhong M.; Wang C.; Pan Z.; Takata T.; Nakabayashi M.; Shibata N.; Li Y.; Sharp I. D.; Kudo A.; Yamada T.; Domen K. Scalable water splitting on particulate photocatalyst sheets with a solar-to-hydrogen energy conversion efficiency exceeding 1%. Nat. Mater. 2016, 15, 611–615. 10.1038/nmat4589. 26950596

[ref49] Wang Q.; Okunaka S.; Tokudome H.; Hisatomi T.; Nakabayashi M.; Shibata N.; Yamada T.; Domen K. Printable Photocatalyst Sheets Incorporating a Transparent Conductive Mediator for Z-Scheme Water Splitting. Joule 2018, 2, 2667–2680. 10.1016/j.joule.2018.08.003.

[ref50] Mortimer R. J. Organic electrochromic materials. Electrochim. Acta 1999, 44, 2971–2981. 10.1016/S0013-4686(99)00046-8.

[ref51] Groenendaal L. B.; Jonas F.; Freitag D.; Pielartzik H.; Reynolds J. R. Poly(3,4-ethylenedioxythiophene) and Its Derivatives: Past, Present, and Future. Adv. Mater. 2000, 12, 481–494. 10.1002/(SICI)1521-4095(200004)12:7<481::AID-ADMA481>3.0.CO;2-C.

[ref52] Heeger A. J. Semiconducting and Metallic Polymers: The Fourth Generation of Polymeric Materials. J. Phys. Chem. B 2001, 105, 8475–8491. 10.1021/jp011611w. 29712324

[ref53] Heinze J.; Frontana-Uribe B. A.; Ludwigs S. Electrochemistry of Conducting Polymerss Persistent Models and New Concepts. Chem. Rev. 2010, 110, 4724–4771. 10.1021/cr900226k. 20557047

[ref54] Iwase A.; Ikeda S.; Kudo A. Efficient Solar Water Oxidation to Oxygen over Mo-doped BiVO_4_ Thin Film Photoanode Prepared by a Facile Aqueous Solution Route. Chem. Lett. 2017, 46, 651–654. 10.1246/cl.170052.

[ref55] Bredas J. L.; Scott J. C.; Yakushi K.; Street G. B. Polarons and bipolarons in polypyrrole: Evolution of the band structure and optical spectrum upon doping. Phys. Rev. B 1984, 30, 1023–1025. 10.1103/PhysRevB.30.1023.

[ref56] Niu G.; Yang S.; Li H.; Yi J.; Wang M.; Lv X.; Zhong J. Electrodeposition of Cu-Ga Precursor Layer from Deep Eutectic Solvent for CuGaS_2_ Solar Energy Thin Film. J. Electrochem. Soc. 2014, 161, D333 10.1149/2.050406jes.

[ref57] Nasybulin E.; Wei S.; Kymissis I.; Levon K. Effect of solubilizing agent on properties of poly(3,4-ethylenedioxythiophene) (PEDOT) electrodeposited from aqueous solution. Electrochim. Acta 2012, 78, 638–643. 10.1016/j.electacta.2012.06.083.

[ref58] Cheng Y.; Xiao C.; Mahmoudi B.; Scheer R.; Maijenburg A. W.; Osterloh F. E. Effect of charge selective contacts on the quasi Fermi level splitting of CuGa_3_Se_5_ thin film photocathodes for hydrogen evolution and methylviologen reduction. EES Catal. 2023, 1, 74–83. 10.1039/D2EY00062H.

[ref59] Srinivasan N.; Shiga Y.; Atarashi D.; Sakai E.; Miyauchi M. A PEDOT-coated quantum dot as efficient visible light harvester for photocatalytic hydrogen production. Appl. Catal. B: Environ. 2015, 179, 113–121. 10.1016/j.apcatb.2015.05.007.

[ref60] Lotfi S.; Navaee A.; Salimi A. Light-Driven Photocatalytic Hydrogen Evolution on Spindle-like MoS_x_ Nanostructures Grown on Poly-Salicylic Acid Synthesized through Bipolar Electrochemistry. ACS Sustainable Chem. Eng. 2018, 6, 9784–9792. 10.1021/acssuschemeng.8b00847.

[ref61] Fumagalli F.; Bellani S.; Schreier M.; Leonardi S.; Rojas H. C.; Ghadirzadeh A.; Tullii G.; Savoini A.; Marra G.; Meda L.; Gratzel M.; Lanzani G.; Mayer M. T.; Antognazza M. R.; Di Fonzo F. Hybrid organic-inorganic H_2_-evolving photocathodes: understanding the route towards high performances organic photoelectrochemical water splitting. J. Mater. Chem. A 2016, 4, 2178–2187. 10.1039/C5TA09330A.

[ref62] Chae S. Y.; Lee M.; Je Kim M.; Cho J. H.; Kim B.; Joo O.-S. p-CuInS_2_/n-Polymer Semiconductor Heterojunction for Photoelectrochemical Hydrogen Evolution. ChemSusChem 2020, 13, 6651–6659. 10.1002/cssc.202002123. 33119209

[ref63] Won D. H.; Chung J.; Park S. H.; Kim E.-H.; Woo S. I. Photoelectrochemical production of useful fuels from carbon dioxide on a polypyrrole-coated p-ZnTe photocathode under visible light irradiation. J. Mater. Chem. A 2015, 3, 1089–1095. 10.1039/C4TA05901H.

