# Supplementary material for: Enhancing Photocathodic Performances of Particulate-CuGaS2-Based Photoelectrodes via Conjugation with Conductive Organic Polymers for Efficient Solar-Driven Hydrogen Production and CO2 Reduction
Source: ACS Appl Mater Interfaces. 2024 Jul 2;16(28):36423–32. doi: 10.1021/acsami.4c06083 (PMC11261570; doi:10.1021/acsami.4c06083)
Supplement: Supplementary file 1 — am4c06083_si_001.pdf [file am4c06083_si_001.pdf]

Supporting Information

Enhancing Photocathodic Performances of Particulate-$CuGaS_2$-Based Photoelectrodes via Conjugation with Conductive Organic Polymers for Efficient Solar-Driven Hydrogen Production and CO_2 Reduction

Tomoaki Takayama,[a ‡] Akihide Iwase,[a§] Akihiko Kudo[a, b]*

[a]*Department of Applied Chemistry, Faculty of Science, Tokyo University of Science, 1-3 Kagurazaka, Shinjuku-ku, Tokyo 162-8601, Japan.*

[b]*Tokyo University of Science, Research Institute of Science and Technology, Carbon Value Research Center, Japan.*

[‡]*Present address: His current affiliation is Graduate School of Science and Technology, Division of Materials Science, Nara Institute of Science and Technology, 8916-5 Takayama, Ikoma, Nara 630-0192, Japan.*

[§]*Present address: His current affiliation is Department of Applied Chemistry, School of Science and Technology, Meiji University, Kanagawa 214-8571, Japan.*

Corresponding author: a-kudo@rs.tus.ac.jp

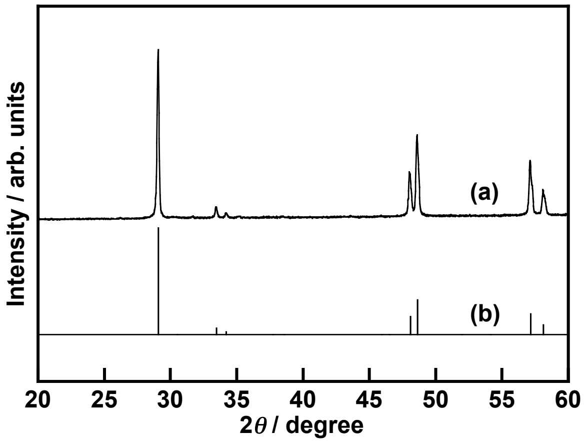

Figure S1. XRD patterns of (a) a $CuGaS_2$ photocatalyst powder and (b) PDF of $CuGaS_2$ (1-85-1574).

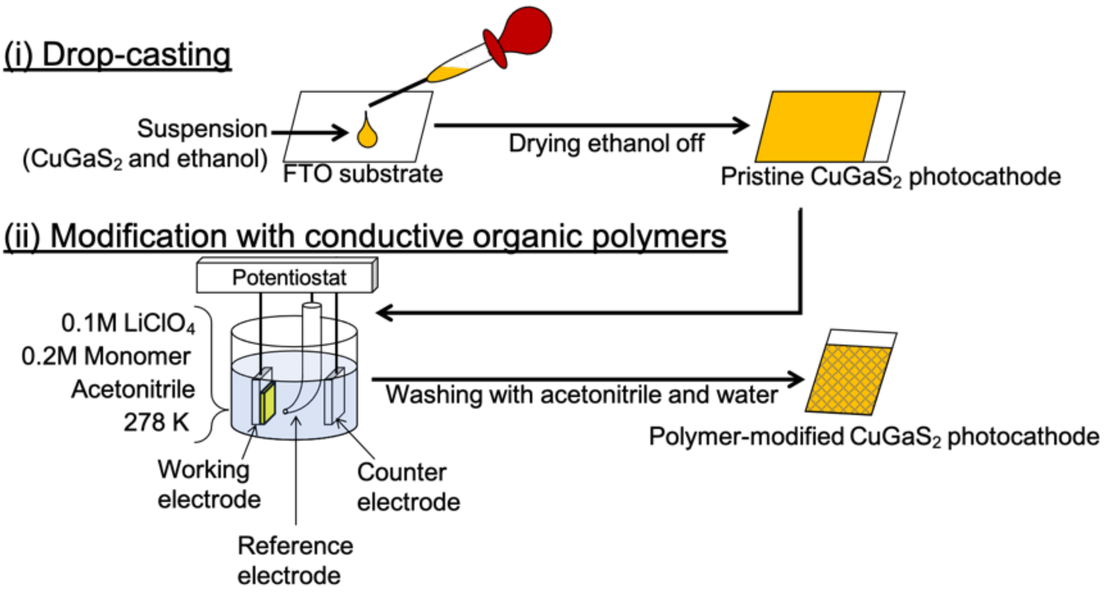

Figure S2. Brief explanation of the preparation procedure of polymer-modified $CuGaS_2$ photocathode.

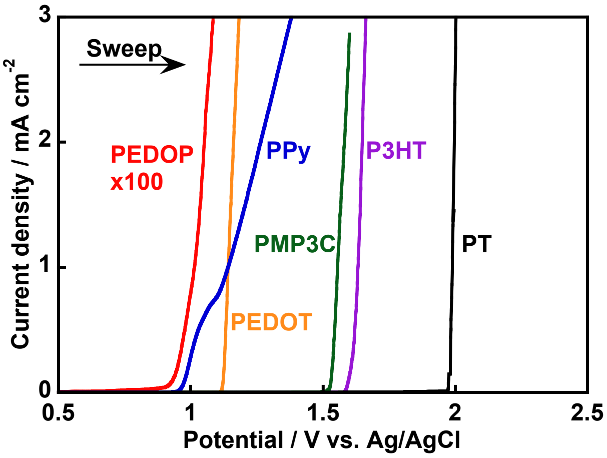

Figure S3. Linear sweep voltammograms of electrochemical oxidative polymerization of various monomers. Electrolyte: 0.1 mol L^{-1} $LiClO_4$ acetonitrile solution saturated with N_2 (1 atm) at 278 K (an ice bath). Sweep rate was 1 mV s^{-1}. The concentrations of the respective monomers are displayed in the experimental section of the main text.

Comments on Figure S3;

The respective linear sweep voltammograms were recorded using bare FTO substrates as the working and counter electrodes. Considering these voltammograms, the applied potential ranges for the electrochemical oxidative polymerization were determined as follows. When a particulate-$CuGaS_2$-based electrode was modified with PPy or PEDOP, +1 or +1.1 V vs. Ag/AgCl was continuously applied to the electrode. On the other hand, PT, PMP3C, P3HT, or PEDOT modified one was prepared through forward and backward sweeps several times in the following potentials; PT (1.5 V to 2.4 V), PMP3C (1.4 V to 1.6 V), P3HT (1.5 V to 2.0 V), PEDOT (0.9 V to 1.3 V). The electrochemical cell was almost the same setup as that shown in Figure S2 (namely, a single 3-electrode cell, no separation of the working, counter, and reference electrodes in the cell).

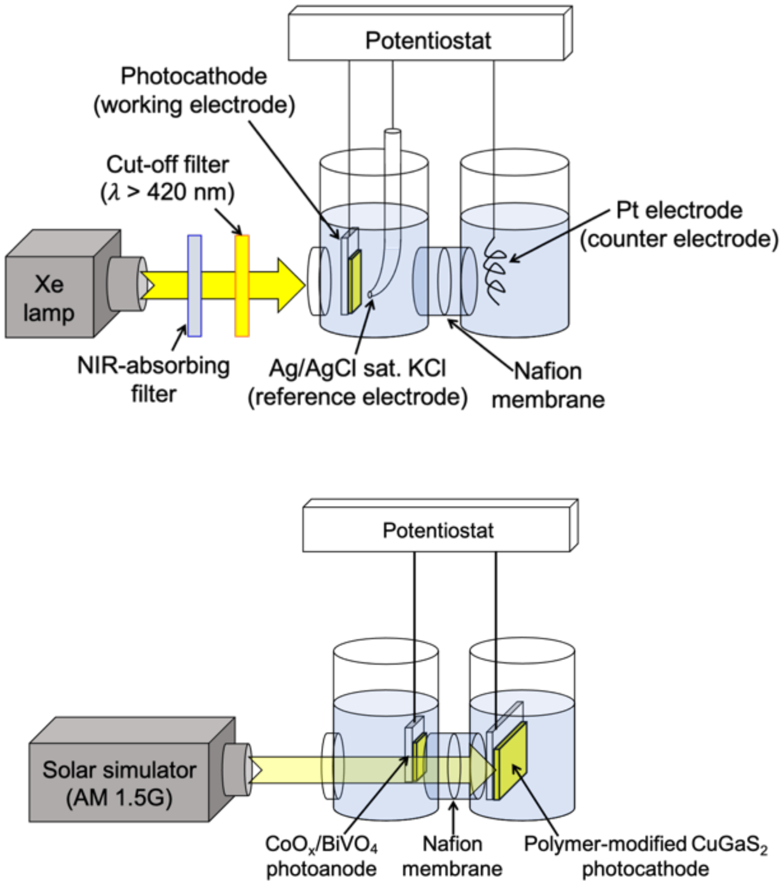

Figure S4. Brief illustration of the setup of the electrochemical cells. Upper illustration indicates a typical three-electrode-type cell, whereas lower one displays two-electrode-type cell. Note that pieces of apparatus for supplying gas and extracting products are omitted.

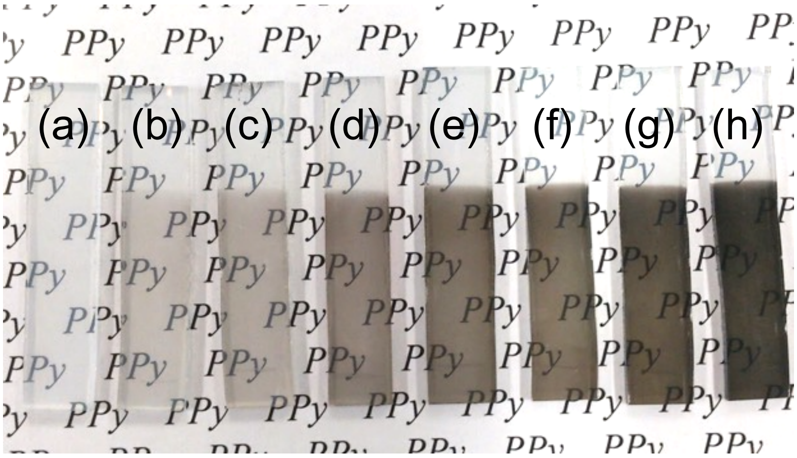

Figure S5. Photographs of (a) a pristine FTO substrate and polypyrrole films on FTO substrates prepared by an electrochemical oxidative polymerization with the electricity of (b) 4 (c) 7, (d) 17, (e) 27, (f) 33, (g) 40, and (h) 50 mC cm^{-2}, respectively.

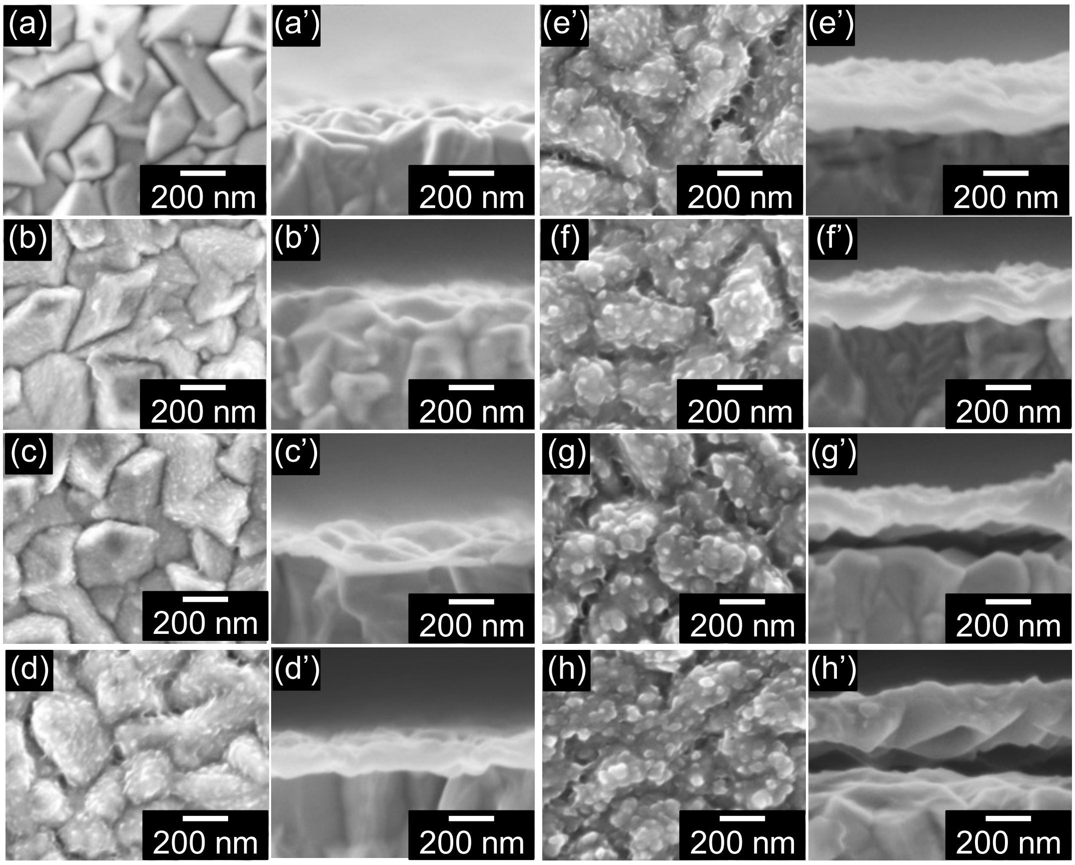

Figure S6. SEM images of (a) a pristine FTO substrate and polypyrrole films on FTO substrates prepared by an electrochemical oxidative polymerization with the electricity of (b) 4 (c) 7, (d) 17, (e) 27, (f) 33, (g) 40, and (h) 50 mC cm^{-2}, respectively. Notations of simple alphabets indicate top sides of the samples, and the notations with prime symbols indicate cross-sections of those.

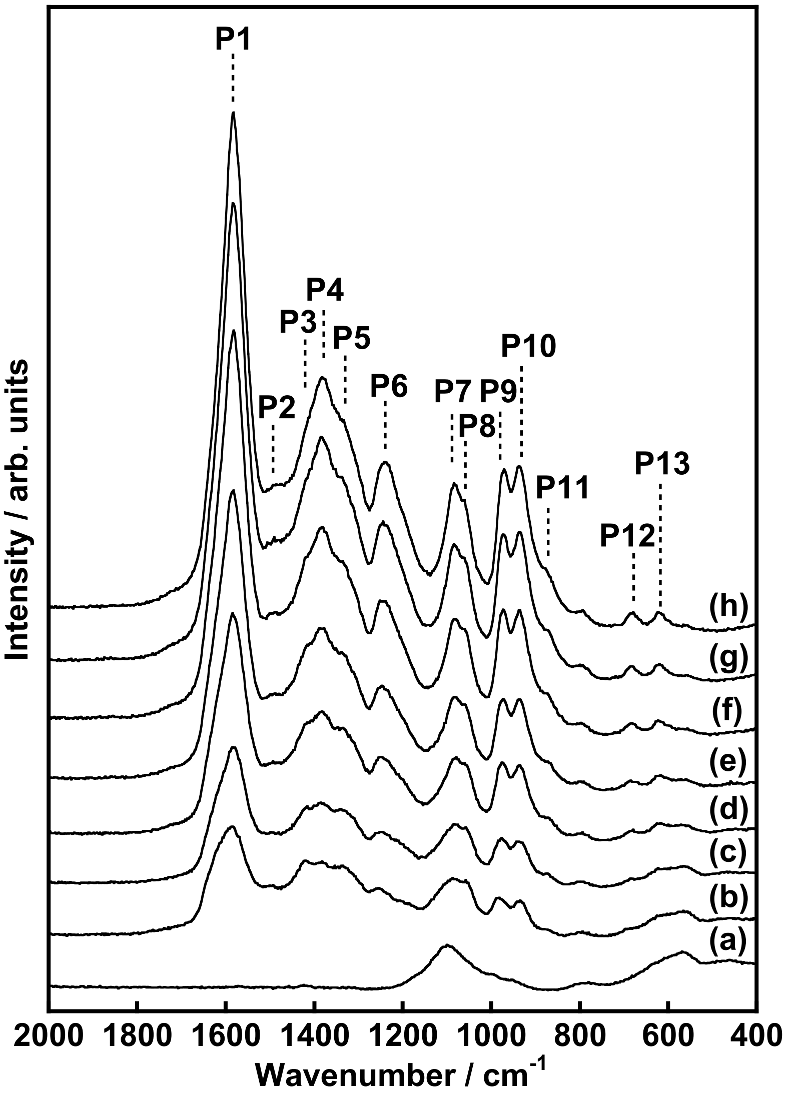

Figure S7. Raman spectra of (a) a pristine FTO substrate and polypyrrole films on FTO substrates prepared by an electrochemical oxidative polymerization with the electricity of (b) 4 (c) 7, (d) 17, (e) 27, (f) 33, (g) 40, and (h) 50 mC cm^{-2}, respectively.

Comments on Figure S7;

Positions of the peaks were summarized as follows; P1: 1583, P2: 1493, P3: 1420, P4: 1378, P5: 1330, P6: 1239, P7: 1088, P8: 1058, P9: 980, P10: 931, P11: 871, P12: 678, P13: 617 cm^{-1}. These peaks were well consistent with the peak positions of polypyrrole reported in the literature[1-3] except for P11.

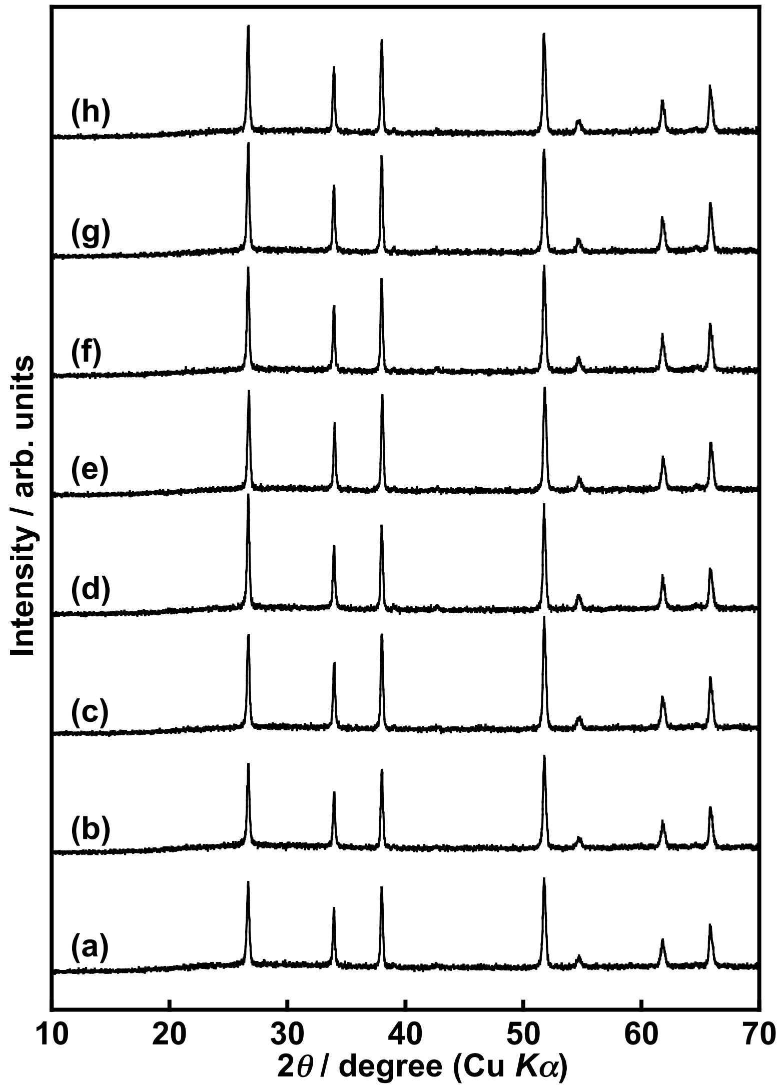

Figure S8. XRD patterns of (a) a pristine FTO substrate and polypyrrole films on FTO substrates prepared by an electrochemical oxidative polymerization with the electricity of (b) 4 (c) 7, (d) 17, (e) 27, (f) 33, (g) 40, and (h) 50 mC cm^{-2}, respectively.

Comments on Figure S8;

There was not any additional peak even when polypyrrole was deposited with the respective electricity. This indicated that the deposited polypyrrole was amorphous.

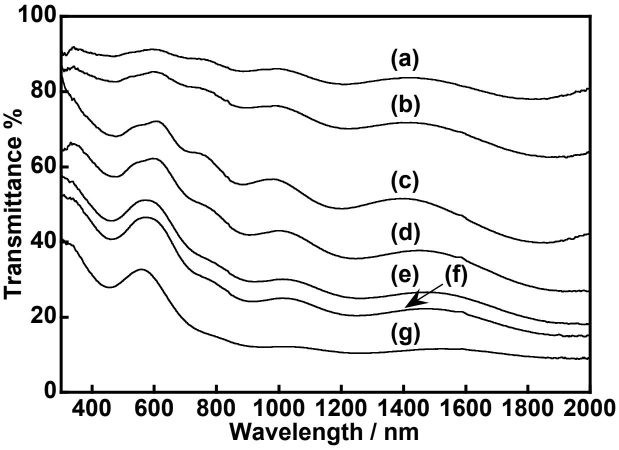

Figure S9. Transmittance spectra of polypyrrole films on FTO substrates prepared by an oxidatively electrochemical polymerization with the electricity of (a) 4 (b) 7, (c) 17, (d) 27, (e) 33, (f) 40, and (g) 50 mC cm^{-2}, respectively.

Comments on Figure S9;

Transmittance was gradually decreased with an increase in the electricity for the polymerization. This was consistent with Figure S5. These structures of spectra resembled to that of a previous report.[4]

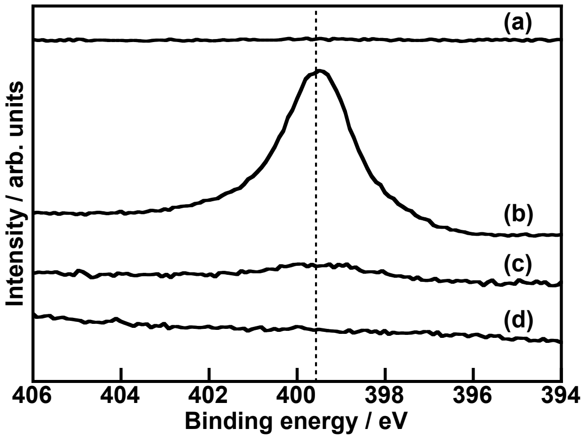

Figure S10. X-ray photoelectron spectra of N 1s signals of (a) a pristine FTO substrate, (b) PPy deposited on a FTO substrate, (c) a $CuGaS_2$ photocathode modified with PPy, and (d) a pristine $CuGaS_2$ photocathode. The amount of electricity of 50 and 90 mC cm^{-2} were used for the PPy polymerization of (b) and (c) samples, respectively. A dotted-line indicates a position of an N 1s signal of –NH– (399.6 eV).[5]

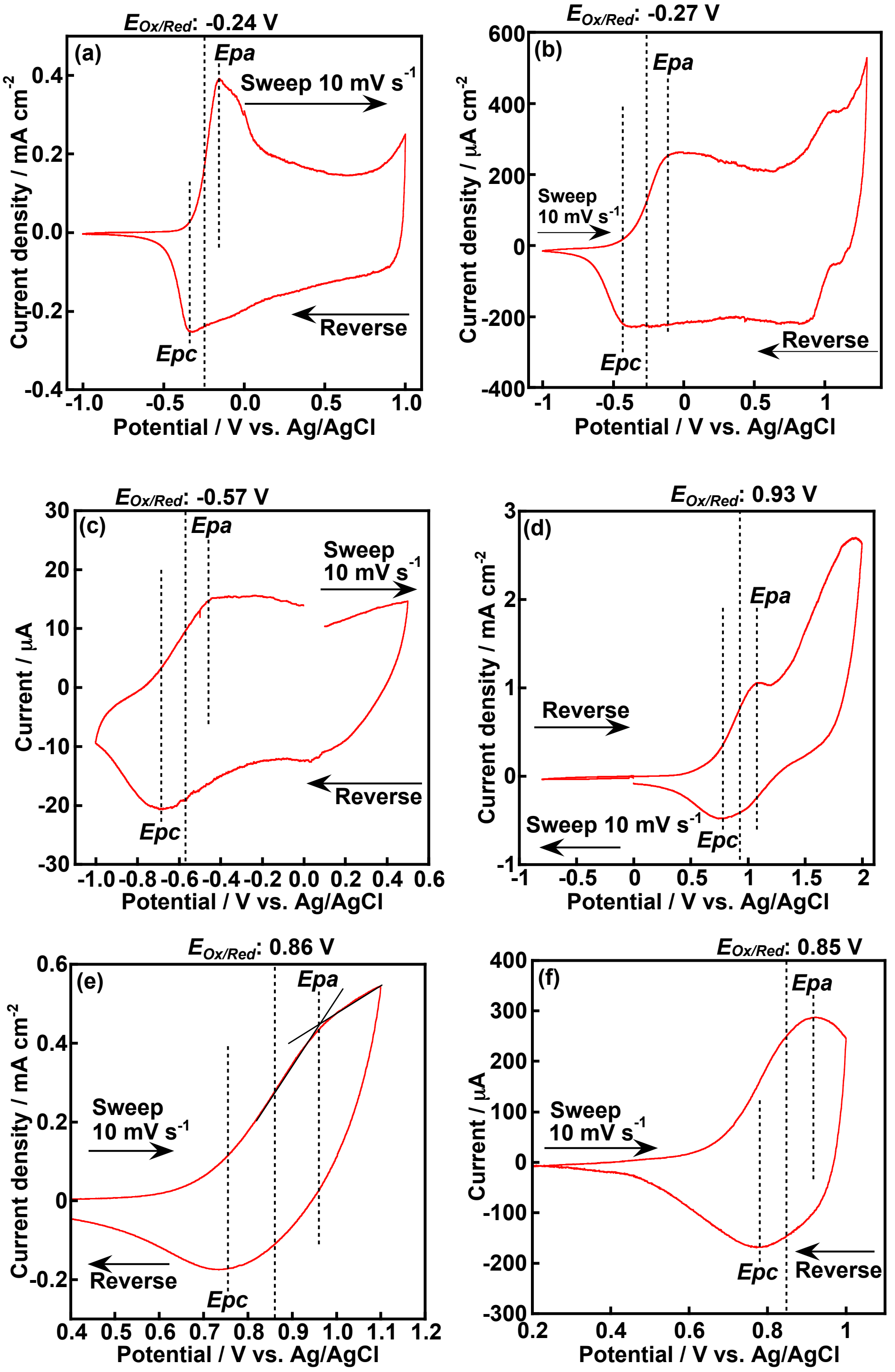

Figure S11. Cyclic voltammograms of (a) PPy, (b) PEDOT, (c) PEDOP, (d) PT, (e) PMP3C, and (f) P3HT. Electrolyte: 0.1 mol L^{-1} $LiClO_4$ acetonitrile solution saturated with N_2 (1 atm) at room temperature.

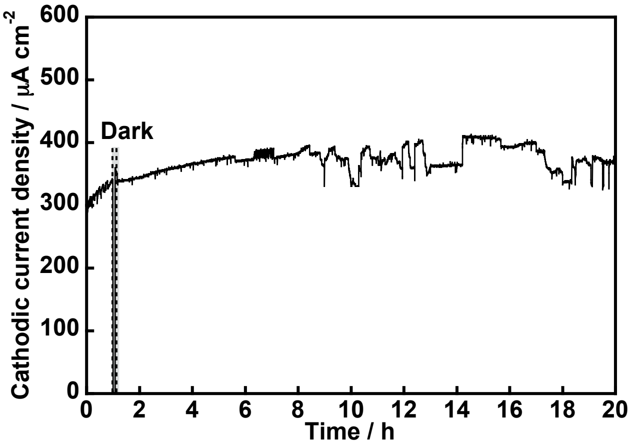

Figure S12. Chronoamperometry of a $CuGaS_2$ photocathode modified with PPy under simulated sunlight irradiation. Electrolyte: 0.1 mol L^{-1} of K_2SO_4 $_{aq.}$ containing a phosphate buffer (pH7) saturated with N_2 gas (1 atm), light source: a solar simulator (AM-1.5G), applied potential: 0 V vs. RHE (pH7). The amount of electricity to prepare PPy was about 50 mC cm^{-2}.

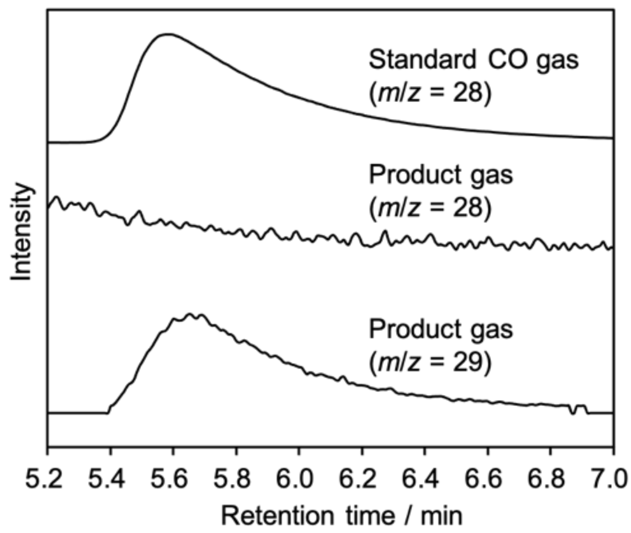

Figure S13. $^{13}CO_2$ reduction over a CGS/PPy photocathode under visible light. Electrolyte: an aqueous K_2SO_4 solution (0.1 mol L^{-1}) saturated with $^{13}CO_2$ gas (1 atm), applied potential: −0.6 V vs. Ag/AgCl, light source: a 300 W Xe-arc lamp with a cut-off filter and an NIR-absorbing filter (λ> 420 nm). The intensity of the standard CO gas was divided by 100.

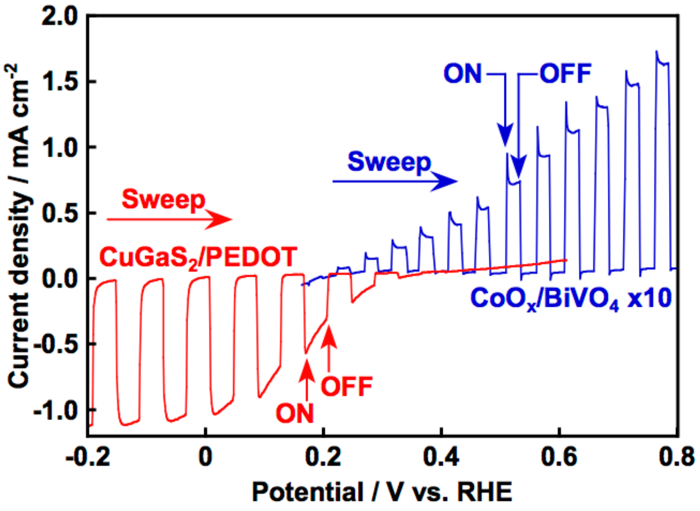

Figure S14. Linear sweep voltammograms of a $CuGaS_2$/PEDOT photocathode and a $CoO_x/BiVO_4$ photoanode under simulated sunlight. Electrolyte: 0.1 mol L^{-1} of an aqueous K_2SO_4 solution with a phosphate buffer saturated with Ar gas (1 atm), light source: a solar simulator (AM-1.5G).

References in the supporting information

1. Liu, Y. C.; Hwang, B. J. Identification of oxidized polypyrrole on Raman spectrum *Synth. Met.* **2000**, *113*, 203−207.
2. Ye, S.; Fang, L.; Qing, X.; Lu, Y. Surface-enhanced Raman scattering study of Ag@PPy nanoparticles *J. Raman Spectrosc.* **2010**, *41*, 1119−1123.
3. Bai, Y.; Xu, Y.; Wang, J.; Gao, M.; Wang, J. Interface Effect on the Electropolymerized Polypyrrole Films with Hollow Micro/Nanohorn Arrays *ACS Appl. Mater. Interfaces* **2014**, *6*, 4693−4704.
4. Bredas, J. L.; Scott, J. C.; Yakushi, K.; Street, G. B. Polarons and bipolarons in polypyrrole: Evolution of the band structure and optical spectrum upon doping *Phys. Rev. B* **1984**, *30*, 1023−1025.
5. Su, N.; Li, H. B.; Yuan, S. J.; Yi, S. P.; Yin, E. Q. Synthesis and characterization of polypyrrole doped with anionic spherical polyelectrolyte brushes *eXPRESS Polymer Letters* **2012**, *6*, 697−705.
